# Genetic variants of *LRRC8C*, *OAS2*, and *CCL25* in the T cell exhaustion-related genes are associated with non-small cell lung cancer survival

**DOI:** 10.3389/fimmu.2024.1455927

**Published:** 2024-10-02

**Authors:** Guojun Lu, Hongliang Liu, Huilin Wang, Xiaozhun Tang, Sheng Luo, Mulong Du, David C. Christiani, Qingyi Wei

**Affiliations:** ^1^ Department of Respiratory Medicine, Nanjing Chest Hospital, Affiliated Nanjing Brain Hospital, Nanjing Medical University, Nanjing, China; ^2^ Duke Cancer Institute, Duke University Medical Center, Durham, NC, United States; ^3^ Department of Population Health Sciences, Duke University School of Medicine, Durham, NC, United States; ^4^ Department of Respiratory Oncology, Guangxi Cancer Hospital, Guangxi Medical University Cancer Hospital, Nanning, China; ^5^ Department of Head and Neck Surgery, Guangxi Cancer Hospital, Guangxi Medical University Cancer Hospital, Nanning, China; ^6^ Department of Biostatistics and Bioinformatics, Duke University School of Medicine, Durham, NC, United States; ^7^ Departments of Environmental Health and Epidemiology, Harvard TH Chan School of Public Health, Boston, MA, United States; ^8^ Department of Medicine, Massachusetts General Hospital, Boston, MA, United States; ^9^ Department of Medicine, Duke University Medical Center, Durham, NC, United States; ^10^ Duke Global Health Institute, Duke University Medical Center, Durham, NC, United States

**Keywords:** non-small cell lung cancer, T cell exhaustion, single-nucleotide polymorphism, prognosis, immunotherapy

## Abstract

**Background:**

T cell exhaustion is a state in which T cells become dysfunctional and is associated with a decreased efficacy of immune checkpoint inhibitors. Lung cancer has the highest mortality among all cancers. However, the roles of genetic variants of the T cell exhaustion-related genes in the prognosis of non-small cell lung cancer (NSCLC) patients has not been reported.

**Methods:**

We conducted a two-stage multivariable Cox proportional hazards regression analysis with two previous genome-wide association study (GWAS) datasets to explore associations between genetic variants in the T cell exhaustion-related genes and survival of NSCLC patients. We also performed expression quantitative trait loci analysis for functional validation of the identified variants.

**Results:**

Of all the 52,103 single nucleotide polymorphisms (SNPs) in 672 T cell exhaustion-related genes, 1,721 SNPs were found to be associated with overall survival (OS) of 1185 NSCLC patients of the discovery GWAS dataset from the Prostate, Lung, Colorectal and Ovarian (PLCO) Cancer Screening Trial, and 125 of these 1,721 SNPs remained significant after validation in an additional independent replication GWAS dataset of 984 patients from the Harvard Lung Cancer Susceptibility (HLCS) Study. In multivariable stepwise Cox model analysis, three independent SNPs (i.e., *LRRC8C* rs10493829 T>C, *OAS2* rs2239193 A>G, and *CCL25* rs3136651 T>A) remained significantly associated with OS with hazards ratios (HRs) of 0.86 (95% confidence interval (CI) = 0.77-0.96, *P* = 0.008), 1.48 (95% CI = 1.18-1.85, *P* < 0.0001) and 0.78 (95% CI = 0.66-0.91, *P* = 0.002), respectively. Further combined analysis for these three SNPs suggested that an unfavorable genotype score was associated with a poor OS and disease-specific survival. Expression quantitative trait loci analysis suggested that the *LRRC8C* rs10493829 C allele was associated with elevated *LRRC8C* mRNA expression levels in normal lymphoblastoid cells, lung tissue, and whole blood.

**Conclusion:**

Our findings suggested that these functional SNPs in the T cell exhaustion-related genes may be prognostic predictors for survival of NSCLC patients, possibly via a mechanism of modulating corresponding gene expression.

## 1 Introduction

Lung cancer is the most commonly diagnosed cancer and the leading cause of cancer mortality worldwide (1). The incidence and mortality rates of lung cancer are gradually declining in the developed countries but still increasing in the developing countries. In 2024, the data from the National Cancer Institute estimate that there will be approximately 234,580 new diagnosed cases and 125,070 deaths from lung cancer in the United States (2). The most prevalent histological form of lung cancer is non-small cell lung cancer (NSCLC) that makes up approximately 85% of all lung cancer cases with high incidence and mortality rates (3). At the time of diagnosis, the majority of NSCLC patients are in advanced stages of the disease, and approximately 60% have some evidence of distant metastases (4). With the improvement in early diagnosis and the advent of new therapeutic methods including immunotherapy, the survival rate of lung cancer continues to improve. However, the 5-year relative survival rate is still poor and even worse in the metastatic setting (5). Therefore, new biomarkers for survival are urgently needed to improve the prognosis of NSCLC.

The emergence of immunotherapy has fundamentally transformed treatment landscape and revolutionized clinical prognosis of solid tumors. Tumor microenvironment (TME) refers to the surrounding environment where diverse cancer cells develop and survive (6). Accumulating evidence indicates that TME plays an important role in the initiation and progression of various cancers (7, 8). Cancer immunotherapy stimulates immune responses and modulates TME to activate T cells to exert an anti-tumor effect (9). Cytotoxic T cells are prototypical anti-tumor immune cell to recognize and eliminate tumor cells that present tumor antigens (10). Moreover, T cells account for the majority of tumor infiltrating lymphocytes that are often correlated with a favorable prognosis and a better response to immunotherapy (11, 12). Despite some impressive progress has enhanced the efficiencies and promising long-term responses, immunotherapy resistance is inevitable for most patients (13). T cell exhaustion, a state in which T cells become dysfunctional as a result of persistent antigenic stimulation within the TME, is one of the potential mechanisms of tumor immunotherapy resistance (14). During chronic infection or cancer, naive T cell first differentiates into T cell exhaustion precursor cells that are unable to fully clear antigens and then into terminal T cell exhaustion that ultimately leads to cell death (15–17). It has been shown that T cell exhaustion is associated with a decreased efficacy of immune-checkpoint inhibitors, including an increased expression of exhaustion markers, decreased effector function, and compromised functionality of T cells (18). Furthermore, T cell exhaustion is associated with immune evasion, disease advancement, and poor survival across multiple cancer types (19). However, up to now, the role of T cell exhaustion-related genes in the survival of NSCLC patients is not fully understood.

Accumulating evidence has suggested that genetic variants, such as single-nucleotide polymorphisms (SNPs) in critical genes, play crucial roles in NSCLC progression and prognosis (20, 21). Genome-wide association studies (GWASs) have successfully identified numerous susceptibility loci for complex diseases (22). However, the roles played by genetic variants of the T cell exhaustion-related genes in survival of NSCLC patients remained unknown. Therefore, in the present study, we tested the hypothesis that genetic variants in the T cell exhaustion-related genes are associated with survival of NSCLC patients in a two-stage analysis using genotyping data from two public GWAS datasets.

## 2 Materials and methods

### 2.1 The discovery dataset

The Prostate, Lung, Colorectal, and Ovarian (PLCO) cancer screening trial is a randomized controlled trial designed to identify the effectiveness of cancer screening for the four cancers (23, 24). Briefly, a total of approximately 155,000 participants, aged 55-74, were recruited from 10 study centers across the United States and enrolled in the PLCO trial that commenced in November 1993 and continued enrolling participants through July 2001. In the discovery stage of the present study, a genotyping dataset of 1185 Caucasian NSCLC patients with the detailed clinical evaluation including lifestyle and medical history was obtained from the PLCO trial for survival analysis. OS and disease-specific survival (DSS) were used as the time-to-event outcomes, and the participants were followed up from the date of diagnosis to the date of last follow-up or death. Whole blood genomic DNA was genotyped using Illumina HumanHap240Sv1.0 and HumanHap550v3.0 platforms (dbGaP accession numbers: phs000093.v2.P2 and phs000336.v1.p1) (25, 26).

### 2.2 The validation dataset

The Harvard University Lung Cancer Susceptibility (HLCS) study recruited pathologically confirmed NSCLC patients from Boston at Massachusetts General Hospital since 1991 (27). The GWAS dataset from the HLCS study of 984 Caucasian NSCLC patients was used as the validation dataset to replicate the findings of the PLCO dataset. In the HLCS study, genomic DNA from blood samples was extracted with the Auto Pure Large Sample Nucleic Acid Purification System (QIAGEN Company, Venlo, Limburg, Netherlands) and genotyped using the Illumina Humanhap610-Quad array. Genotyping data was subsequently used for imputation by using the Minimac4 software based on the 1000 Genomes Project (27).

The use of the two GWAS datasets was approved by the Internal Review Board of Duke University School of Medicine (Project #Pro00054575) and the dbGaP database (Project #6404). The comparison of clinical characteristics between the PLCO trial and the HLCS study is shown in **Supplementary Table 1**.

### 2.3 Gene selection and SNP imputation

The list of T cell exhaustion-related genes was obtained from a previous study (28). After the removal of 11 genes on the X chromosome, 672 remaining genes were considered candidate genes for further analyses (**Supplementary Table 2**) and used for imputation with the Minimac4 and the 1000 Genomes Project (phase 3) dataset. Then, we extracted SNPs located in these genes and their ±2 kb flanking regions by the following criteria: *r* ^2^ ≥ 0.3 (**Supplementary Figure 1**), a minor allele frequency (MAF) ≥ 0.05, an individual call rate ≥ 95%, and the Hardy-Weinberg equilibrium (HWE) ≥ 1 × 10^−5^. As a result, a total of 52,103 candidate SNPs (6,526 genotyped and 45,577 imputed) were selected from the PLCO trial.

### 2.4 Statistical analyses

In the single-locus analysis, we performed a multivariable Cox proportional hazards regression analysis using the R package GenABEL to estimate associations between 52,103 candidate SNPs and NSCLC survival in the PLCO trial with an additive genetic model (29). The Cox analysis was adjusted for available covariates including age, sex, smoking status, histologic subtype, tumor stage, chemotherapy, radiotherapy, surgery, and the top four of the 10 principal components (PCs) in the PLCO dataset (**Supplementary Table 3**). We chose OS as the primary endpoint and also assessed DSS as an endpoint in the survival analysis. In consideration of the high linkage disequilibrium (LD) among these imputed SNPs, we applied Bayesian false discovery probability (BFDP) with a cutoff value of 0.80 for multiple testing corrections to filter the probability of potential false-positive results (30, 31). Moreover, we assigned a prior probability of 0.10 to detect an upper bound of 3.0 for an association with adverse genotypes or minor alleles of each SNP with *P* < 0.05.

The significant SNPs in the PLCO dataset were then validated with the HLCS dataset using a multivariable Cox regression model. The combination of results from both PLCO trial and HLCS study was also performed in the classical inverse variance weighted meta-analysis, in which Cochran’s Q-test and the heterogeneity statistic (*I^2^*) were used to assess the inter-study heterogeneity and to determine the appropriate model. Because no inter-study heterogeneity was found (Q-test *P*-value > 0.10 and the *I* ^2^ < 50%), the combined meta-analysis was conducted with a fixed-effects model.

Since many SNPs inherit together through disequilibrium and thus may provide redundant information, tagger SNPs were selected to represent groups of the correlated SNPs to reduce both redundancy and the number of statistical tests to be performed. In LD analysis (*r* ^2^ < 0.8) using Haploview 4.1 with data from the 1000 Genomes Project (32), potential functions of these SNPs were predicted with two online bioinformatics tools, RegulomeDB (http://www.regulomedb.org/) and HaploReg v4.2 (https://pubs.broadinstitute.org/mammals/haploreg/haploreg.php) (33, 34). Subsequently, a multivariable stepwise Cox regression model with adjustment for demographic and clinical variables, the top four PCs, as well as 54 previously published SNPs from the same PLCO GWAS dataset, was performed to identify the associations between independent SNPs and NSCLC survival. Manhattan plots and regional association plots were generated using Haploview4.1 and Locus Zoom (http://http://locuszoom.sph.umich.edu) respectively to visualize these identified SNPs (35).

Subsequently, the unfavorable genotypes of identified SNPs were combined to evaluate their cumulative effects on NSCLC survival. Stratified analysis by subgroups was performed to calculate the inter-study heterogeneity and possible effect modification or interaction. A survival prediction model, constructed using the receiver operating characteristic (ROC) curves and time-dependent area under the curve (AUC) with R (version 3.6.3) package “Survival” and “time ROC”, was employed to assess the prediction accuracy of the clinical and genetic variables on NSCLC survival (36). To evaluate the genotype-phenotype associations of identified SNPs with the corresponding mRNA expression levels, expression quantitative trait loci (eQTL) analyses with a linear regression model were performed using data from two sources: normal lymphoblastoid cells from 373 European descendants in the 1,000 Genomes Project and the genotype-tissue expression (GTEx) project (including 515 normal lung tissues and 670 whole blood samples) (https://www.gtexportal.org/home, V8) (37).

Finally, we used the XIANTAO (https://www.xiantaozi.com) online data analysis tool that helped analyze cancer omics data of the Cancer Genome Atlas (TCGA) database. Here, we used XIANTAO to compare the differences of mRNA expression levels in NSCLC using paired or unpaired *t*-tests. The online survival analysis platform Kaplan-Meier (http://kmplot.com/analysis/) was also used to assess the correlation between the corresponding mRNA expression levels and the probability of NSCLC survival (38). All statistical analyses were performed using the SAS software (Version 9.4, SAS Institute, Cary, NC, USA), unless specified otherwise.

## 3 Results

### 3.1 Associations between SNPs in the T cell exhaustion-related genes and NSCLC survival

**Figure 1:**
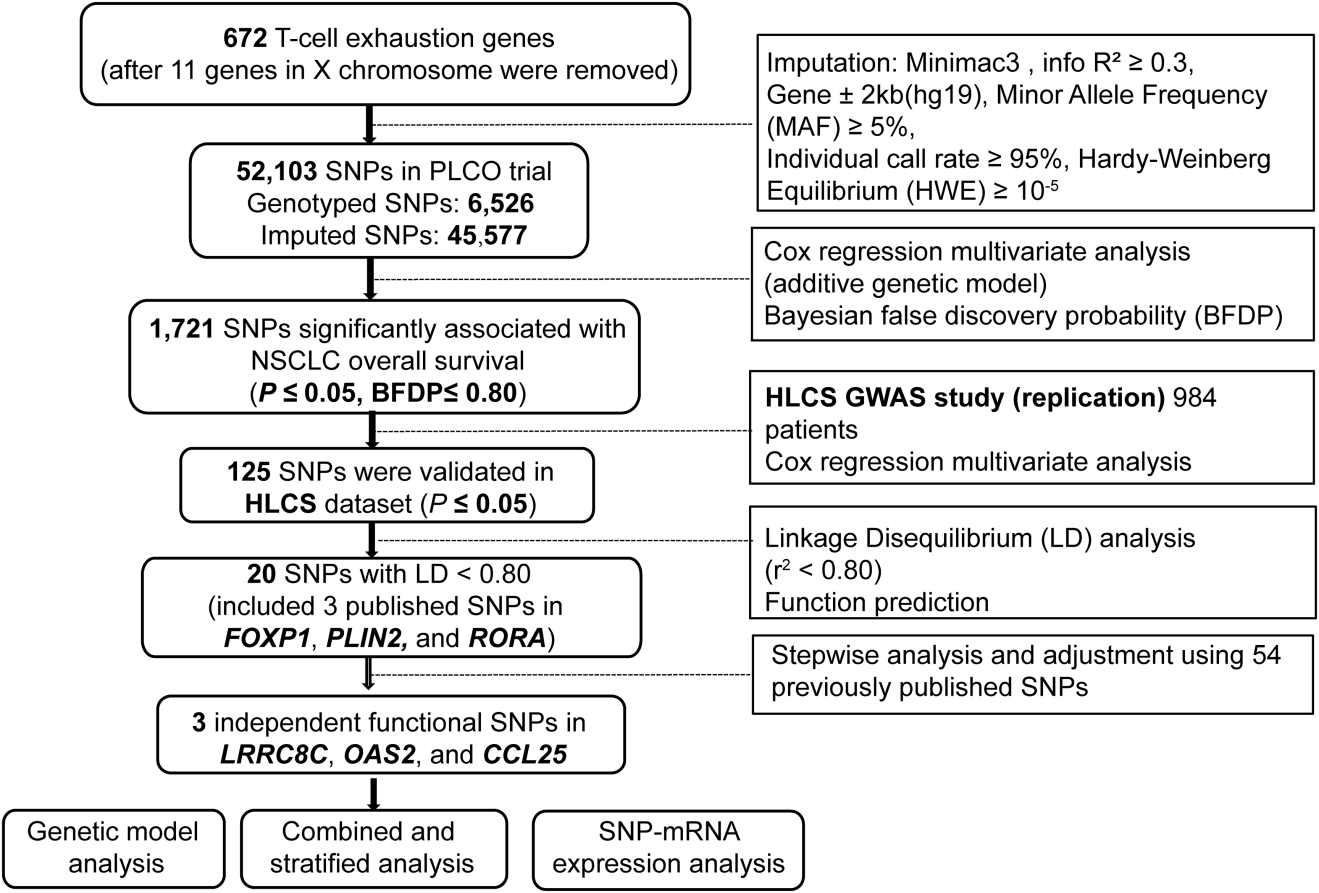
The flowchart of the present study.

In the analysis, we used 1185 NSCLC patients from the PLCO trial and 984 NSCLC patients from the HLCS study. The flow chart of the present study is presented in **Figure 1**. In the discovery after the BFDP correction, we found that 1,721 SNPs (250 genotyped and 1,471 imputed) out of the 52,103 SNPs in the 672 T cell exhaustion-related genes were significantly associated with NSCLC OS (*P* ≤ 0.05). These SNPs were then used for validation with the dataset of the HLCS study, in which 125 SNPs in 12 genes remained significant, five genes (*MET*, *PLIN2*, *OAS2*, *IRF9*, and *PRKCH*) had only one SNP, and the other seven genes had 120 SNPs, of which 15 SNPs (**Supplementary Figure 2**) together with the other five SNPs were selected as the tagger SNPs. Biological function prediction of these SNPs was conducted with two online bioinformatics tools of the HaploReg and RegulomeDB projects. As shown in **Supplementary Table 4**, most SNPs were located in the intronic region of their genes and associated with enhancer histone marks and protein motifs alteration.

### 3.2 Identification of associations between independent SNPs and NSCLC survival in the PLCO trial

**Figure 2:**
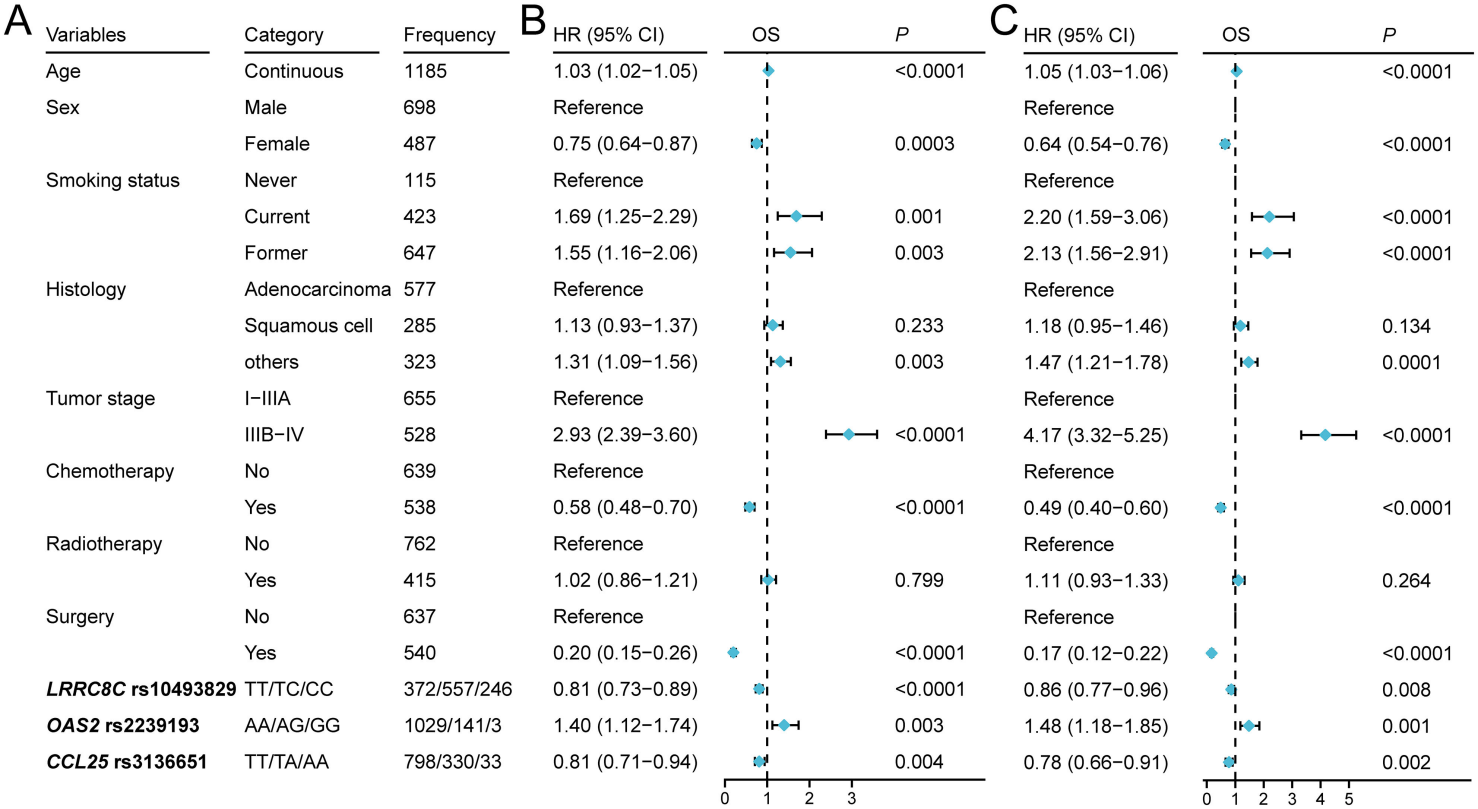
Three independent SNPs in a multivariate Cox proportional hazards regression analysis. **(A)** The characteristics of three SNPs and other covariates. **(B)** Forest map indicated the result of stepwise analysis including age, sex, smoking status, tumor stage, histology, chemotherapy, radiotherapy, surgery, PC1, PC2, PC3, PC4, and SNPs. **(C)** Forest map indicated the result of post-stepwise analysis with adjustment using 54 published SNPs for NSCLC in the same PLCO genotyping dataset: rs779901, rs3806116, rs199731120, rs10794069, rs1732793, rs225390, rs3788142, rs73049469, rs35970494, rs225388, rs7553295, rs1279590, rs73534533, rs677844, rs4978754, rs1555195, rs11660748, rs73440898, rs13040574, rs469783, rs36071574, rs7242481, rs1049493, rs1801701, rs35859010, rs1833970, rs254315, rs425904, rs35385129, rs4487030, rs60571065, rs13213007, rs115613985, rs9673682, rs2011404, rs7867814, rs2547235, rs4733124, rs11787670, rs67715745, rs922782, rs4150236, rs116454384, rs9384742, rs9825224, rs261083, rs76744140, rs6939623, rs113181986, rs2568847, rs11225211, rs10841847, rs2519996, and rs36215.

**Figure 3:**
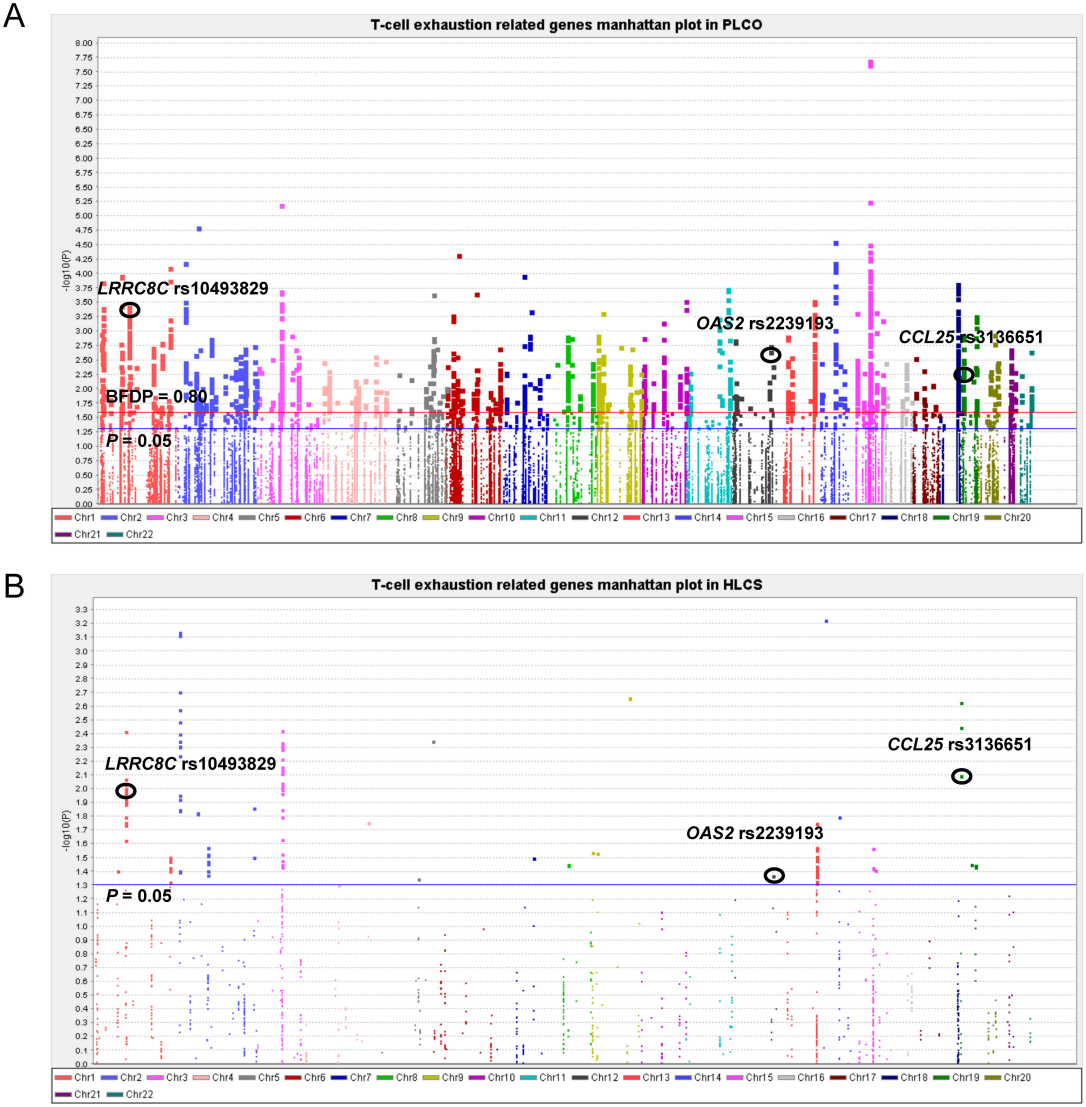
Manhattan plot in the PLCO trial and HLCS study. **(A)** Manhattan plot for 52,103 SNPs of T-cell exhaustion related genes in the PLCO trial. **(B)** Manhattan plot for 1,721 SNPs of T-cell exhaustion-related genes in the HLCS study. The blue horizontal line indicates *P* = 0.05 and the red line indicates BFDP = 0.80.

**Figure 4:**
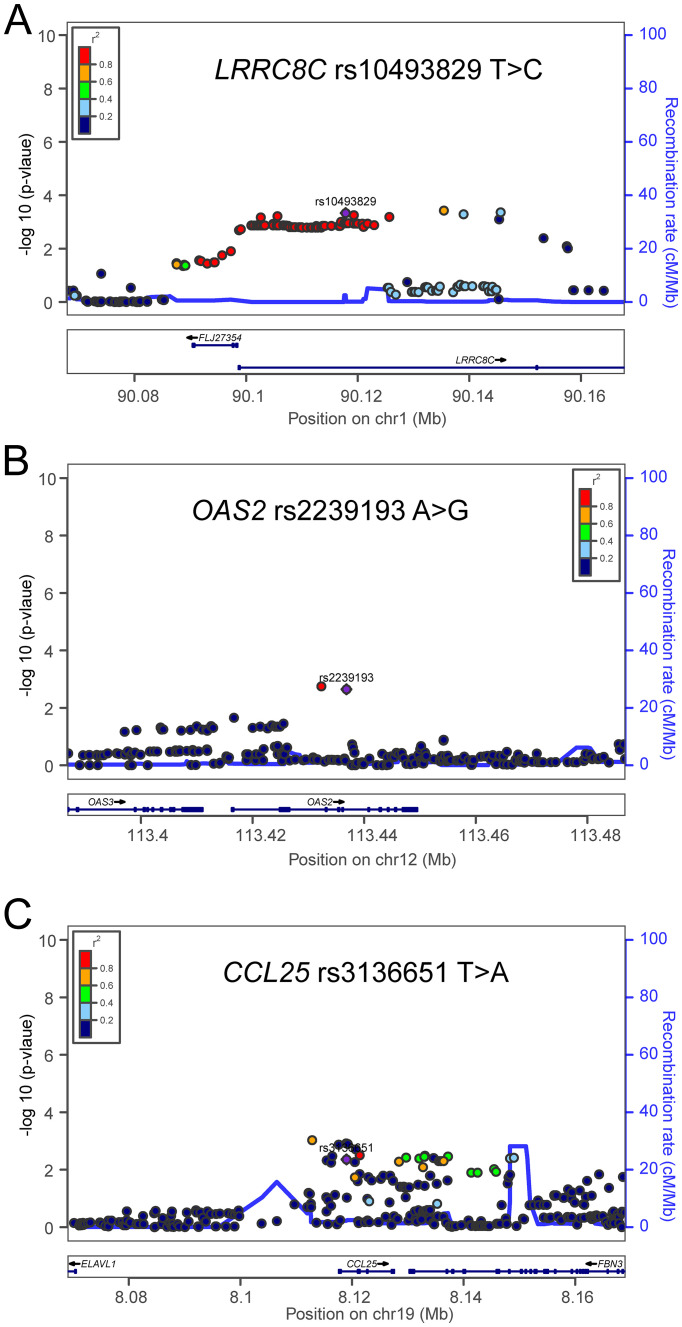
Regional association plots for the three independent SNPs in the T-cell exhaustion related genes. Regional association plots included 50kb up or downstream of **(A)** *LRRC8C*, **(B)** *OAS2*, and **(C)** *CCL25*. Data points are colored according to the level of linkage disequilibrium of each pair of SNPs based on the hg19/1000 Genomes European population. The left-hand y-axis shows the association *P*-value of individual SNPs in the discovery dataset, which is plotted as -log10 (P) against chromosomal base-pair position. The right-hand y-axis shows the recombination rate estimated from HapMap Data Rel 22/phase II European population.

To assess the effect of independent SNPs on NSCLC survival in the PLCO trial, we first performed stepwise multivariable Cox regression analysis. Then, SNPs that remained significant were put into a post-stepwise multivariable Cox model with adjustment for 54 previously reported SNPs in the same PLCO trial. Finally, three independent SNPs (*LRRC8C* rs10493829 T>C, *OAS2* rs2239193 A>G, and *CCL25* rs3136651 T>A) remained significantly associated with NSCLC OS (*P* = 0.008, *P* = 0.001, and *P* = 0.002, respectively) (**Figure 2**). Moreover, the results of meta-analysis for these three identified SNPs across the PLCO trial and HLCS study are presented in **Table 1**, and no heterogeneity was observed. We also depicted the locations of these three significant SNPs in Manhattan plots (**Figure 3**) and regional association plots (**Figure 4**).

**Table 1:** Associations of three significant SNPs with of NSCLC overall survival in both discovery and validation datasets from two published GWASs.

SNPs	Allele ^a^	Gene	PLCO (n=1,185)			HLCS (n=984)			Meta-analysis			
			EAF	HR (95% CI) ^b^	*P* ^b^	EAF	HR (95% CI) ^C^	*P* ^C^	*P*_het_ ^d^	*I* ^2^	HR (95% CI) ^e^	*P* ^e^
rs10493829	T>C	*LRRC8C*	0.44	0.84 (0.76-0.93)	0.001	0.47	0.87 (0.79-0.97)	0.011	0.597	0	0.86 (0.80-0.92)	1.58x10^-5^
rs2239193	A>G	*OAS2*	0.06	1.38 (1.12-1.69)	0.002	0.06	1.23 (1.01-1.50)	0.043	0.425	0	1.30 (1.13-1.50)	3.41x10^-4^
rs3136651	T>A	*CCL25*	0.17	0.82 (0.72-0.94)	0.004	0.14	0.81 (0.70-0.95)	0.008	0.935	0	0.82 (0.74-0.90)	9.47x10^-5^

SNPs, single-nucleotide polymorphisms; NSCLC, non-small cell lung cancer; GWAS, genome-wide association study; PLCO, the Prostate, Lung, Colorectal and Ovarian cancer screening trial; HLCS, Harvard Lung Cancer Susceptibility Study; EAF, effect allele frequency; HR, hazards ratio; CI, confidence interval.

a Reference/effect allele.

b Adjusted for age, sex, stage, histology, smoking status, chemotherapy, radiotherapy, surgery, identified SNPs, PC1, PC2, PC3, and PC4.

c Adjusted for age, sex, stage, histology, smoking status, chemotherapy, radiotherapy, surgery, PC1, PC2, and PC3.

d P_het_: P value for heterogeneity by Cochrane’s Q test.

e Meta-analysis in the fix-effects model.

As shown in **Table 2**, the *LRRC8C* rs10493829 C allele and *CCL25* rs3136651 A allele were associated with better NSCLC OS (*P*_trend_ = 0.0005 and 0.003, respectively) and DSS (*P*_trend_ = 0.0001 and 0.009, respectively), while the *OAS2* rs2239193 G allele was associated with poor NSCLC OS and DSS (*P*_trend_ = 0.002 for both). In a dominant genetic model, compared with the reference genotype, NSCLC patients had a poor survival associated with *LRRC8C* rs10493829 TT (OS: HR = 1.18, 95% CI = 1.01-1.37, *P* = 0.035), *OAS2* rs2239193 AG+GG (OS: HR = 1.38, 95% CI = 1.11-1.70, *P* = 0.003; DSS: HR = 1.43, 95% CI = 1.15-1.78, *P* = 0.001) and *CCL25* rs3136651 TT (OS: HR = 1.20, 95% CI = 1.02-1.40, *P* = 0.024; DSS: HR = 1.19, 95% CI = 1.01-1.40, *P* = 0.037). As a result, those risk genotypes were considered unfavorable genotypes. We also depicted these results in Kaplan-Meier survival curves (**Supplementary Figure 3**). These results suggested the three SNPs were independently associated with NSCLC survival.

**Table 2:** Associations between the NUGs of three independent SNPs with NSCLC OS and DSS in the PLCO Trial.

Genotype	Frequency	OS ^a^			DSS ^a^		
		Death (%)	HR (95% CI)	*P*	Death (%)	HR (95% CI)	*P*
*LRRC8C* rs10493829 T>C ^b^							
TT	372	257 (60.09)	1.00	–	229 (61.56)	1.00	–
TC	557	377 (67.68)	0.94(0.80-1.10)	0.442	337 (60.50)	0.94 (0.80-1.12)	0.502
CC	246	155 (63.01)	0.69 (0.56-0.84)	0.0003	143 (58.13)	0.72 (0.58-0.89)	0.003
Trend test				0.0005			0.0001
Dominant							
TT	372	257 (60.09)	1.00	–	229 (61.56)	1.00	–
TC+CC	803	532 (66.25)	0.85 (0.73-0.99)	0.035	480 (59.78)	0.87 (0.74-1.02)	0.075
Or reverse							
TC+CC	803	532 (66.25)	1.00	–	480 (59.78)	1.00	–
**TT**	**372**	**257 (60.09)**	**1.18 (1.01-1.37)**	**0.035**	**229 (61.56)**	**1.16 (0.99-1.35)**	**0.075**
*OAS2* rs2239193 A>G ^c^							
AA	1029	682 (66.28)	1.00	–	609 (59.18)	1.00	–
AG	141	103 (73.05)	1.36 (1.10-1.68)	0.004	97 (68.79)	1.43 (1.15-1.78)	0.002
GG	3	2 (66.67)	2.62 (0.64-10.75)	0.180	1 (33.33)	1.76 (0.24-12.69)	0.575
Trend test				0.002			0.002
Dominant							
AA	1029	682 (66.28)	1.00	–	609 (59.18)	1.00	–
**AG+GG**	**144**	**105 (72.92)**	**1.38 (1.11-1.70)**	**0.003**	**98 (68.06)**	**1.43 (1.15-1.78)**	**0.001**
*CCL25* rs3136651 T>A ^d^							
TT	798	538 (67.42)	1.00	–	485 (60.78)	1.00	–
TA	330	224 (67.88)	0.89 (0.76-1.04)	0.149	199 (60.30)	0.88 (0.75-1.04)	0.139
AA	33	15 (45.45)	0.42 (0.25-0.71)	0.001	14 (42.42)	0.49 (0.29-0.85)	0.011
Trend test				0.003			0.009
Dominant							
TT	798	538 (67.42)	1.00	–	485 (60.78)	1.00	–
TA+AA **Or reverse**	363	239 (65.84)	0.84 (0.72-0.98)	0.024	213 (58.68)	0.84 (0.72-0.99)	0.037
TA+AA	363	239 (65.84)	1.00		213 (58.68)	1.00	
**TT**	**798**	**538 (67.42)**	**1.20 (1.02- 1.40)**	**0.024**	**485 (60.78)**	**1.19 (1.01-1.40)**	**0.037**
NUG ^e^							
0	226	143 (63.27)	1.00	–	133 (58.85)	1.00	–
1	590	393 (66.61)	1.13 (0.93-1.38)	0.206	342 (57.97)	1.05 (0.86-1.29)	0.616
2	312	217 (69.55)	1.45 (1.17-1.79)	0.0007	199 (63.78)	1.40 (1.12-1.75)	0.003
3	31	22 (70.97)	1.80 (1.12-2.89)	0.015	22 (70.97)	1.95 (1.21-3.13)	0.006
Trend test				<0.0001			0.0002
Dichotomized NUG							
0-1	816	536 (65.69)	1.00	–	475 (58.21)	1.00	–
2-3	343	239 (69.68)	1.35 (1.15-1.58)	0.0002	221 (64.43)	1.39 (1.18-1.63)	<0.0001

SNP, single nucleotide polymorphism; NSCLC, non-small cell lung cancer; OS, overall survival; DSS, disease-specific survival. PLCO, Prostate, Lung, Colorectal and Ovarian cancer screening trial; HR, hazards ratio; CI, confidence interval; NPA, number of protective alleles.

a Adjusted for age, sex, smoking status, histology, tumor stage, chemotherapy, surgery, radiotherapy and principal components.

b 10 with missing data were excluded.

c 12 with missing data were excluded.

d 24 with missing data were excluded.

e Unfavorable genotypes were LRRC8C rs10493829 TT, OAS2 rs2239193 AG+GG, and CCL25 rs3136651 TT and their results are in bold.

### 3.3 Combined analyses of the associations between the three independent SNPs and NSCLC survival in the PLCO dataset

**Figure 5:**
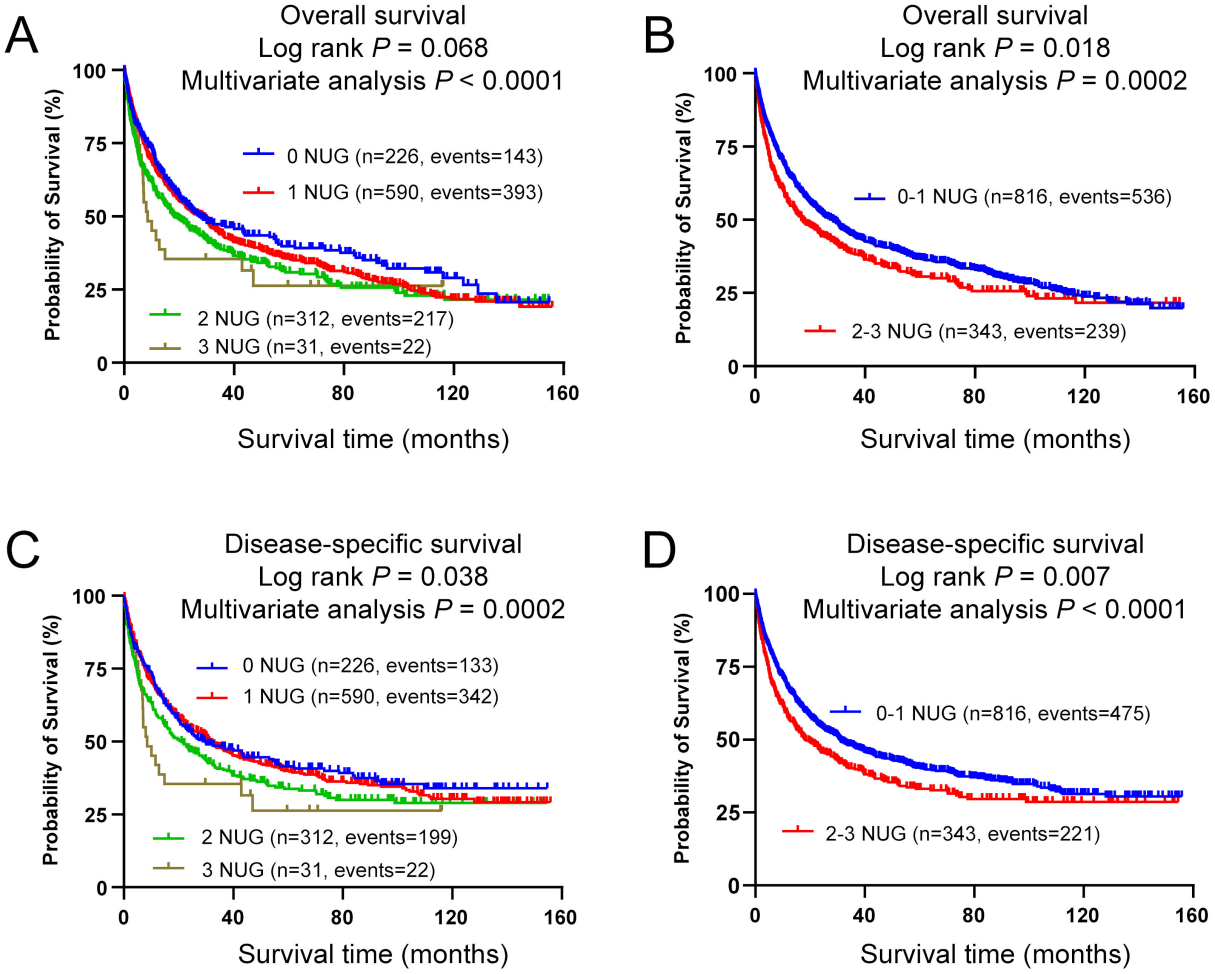
Prediction of survival with combined unfavorable genotypes. Kaplan-Meier survival curves in the PLCO dataset for **(A)** OS with the combined unfavorable genotypes, **(B)** OS with the dichotomized groups of the NUGs, **(C)** DSS with the combined unfavorable genotypes, **(D)** DSS with dichotomized groups of the NUGs. Unfavorable genotypes were *LRRC8C* rs10493829 TT, *OAS2* rs2239193 AG+GG, and *CCL25* rs3136651 TT.

To evaluate accumulative effect of the three independent SNPs on NSCLC survival, we combined their unfavorable genotypes (i.e., *LRRC8C* rs10493829 TT, *OAS2* rs2239193 AG+GG, and *CCL25* rs3136651 TT) into a genetic score that was used to categorize all NSCLC patients into four groups (i.e., 0, 1, 2, and 3) by the number of their unfavorable genotypes (NUG). The results presented in **Table 2** suggested that an increased NUG score was associated with a poorer NSCLC survival for both OS (*P*_trend_ < 0.0001) and DSS (*P*_trend_ = 0.0002) in the multivariable Cox model. We further dichotomized all NSCLC patients into two groups: 0-1 and 2-3 NUGs. Compared with the 0-1 NUG group, the 2-3 NUG group had a significantly poorer OS (HR = 1.35, 95% CI = 1.15-1.58, *P* = 0.0002) and DSS (HR = 1.39, 95% CI = 1.18-1.63, *P* < 0.0001). Furthermore, we also depicted these results with Kaplan-Meier survival curves (**Figures 5A–D**). To sum up, these combined analyses indicated that an increased NUG score was associated with a poorer NSCLC survival.

### 3.4 Stratified analyses for the effect of NUG on NSCLC survival in the PLCO dataset

To explore whether the effect of NUG on NSCLC survival was influenced by other clinical covariates, we performed stratified analyses by age, sex, smoking status, histology, tumor stage, chemotherapy, radiotherapy, and surgery in the PLCO trial. As shown in **Supplementary Table 5**, for the effect on NSCLC OS and DSS, there were no significant interactions of NUG with age, smoking status, histology, tumor stage on DSS, chemotherapy, radiotherapy, and surgery (all *P*_inter_ > 0.05). However, the interaction of NUG with sex (OS: *P* = 0.0004 and DSS: *P* = 0.0005) and tumor stage (*P* = 0.034) was statistically significant (*P*_inter_ < 0.05).

### 3.5 Time-dependent AUC and ROC curves to predict NSCLC survival for the three independent SNPs

To further assess the predictive value of these three independent SNPs, we performed the time-dependent AUC and ROC curves for OS and DSS at the 12^th^, 36^th^, and 60^th^ month with the clinical variables in the PLCO trial. Time-dependent AUC for OS and DSS are listed in **Supplementary Figures 4A, B**. With the addition of the three SNPs to the predictive model, there were no significantly improved NSCLC survival curves at 12^th^ for OS (*P* = 0.087) and DSS (*P* = 0.065) (**Supplementary Figures 4C, D**), 36^th^ for OS (*P* = 0.607) and DSS (*P* = 0.329) (**Supplementary Figures 4E, F**), 60^th^ for OS (*P* = 0.090) (**Supplementary Figure 4G**). However, the predictive performance of AUC curves at the 60^th^ month for DSS was significantly improved (*P* = 0.045) (**Supplementary Figure 4H**). These data suggested that the addition of the three SNPs to the predictive model could only improve AUC at the 5-year DSS.

### 3.6 The result of eQTL analyses

**Figure 6:**
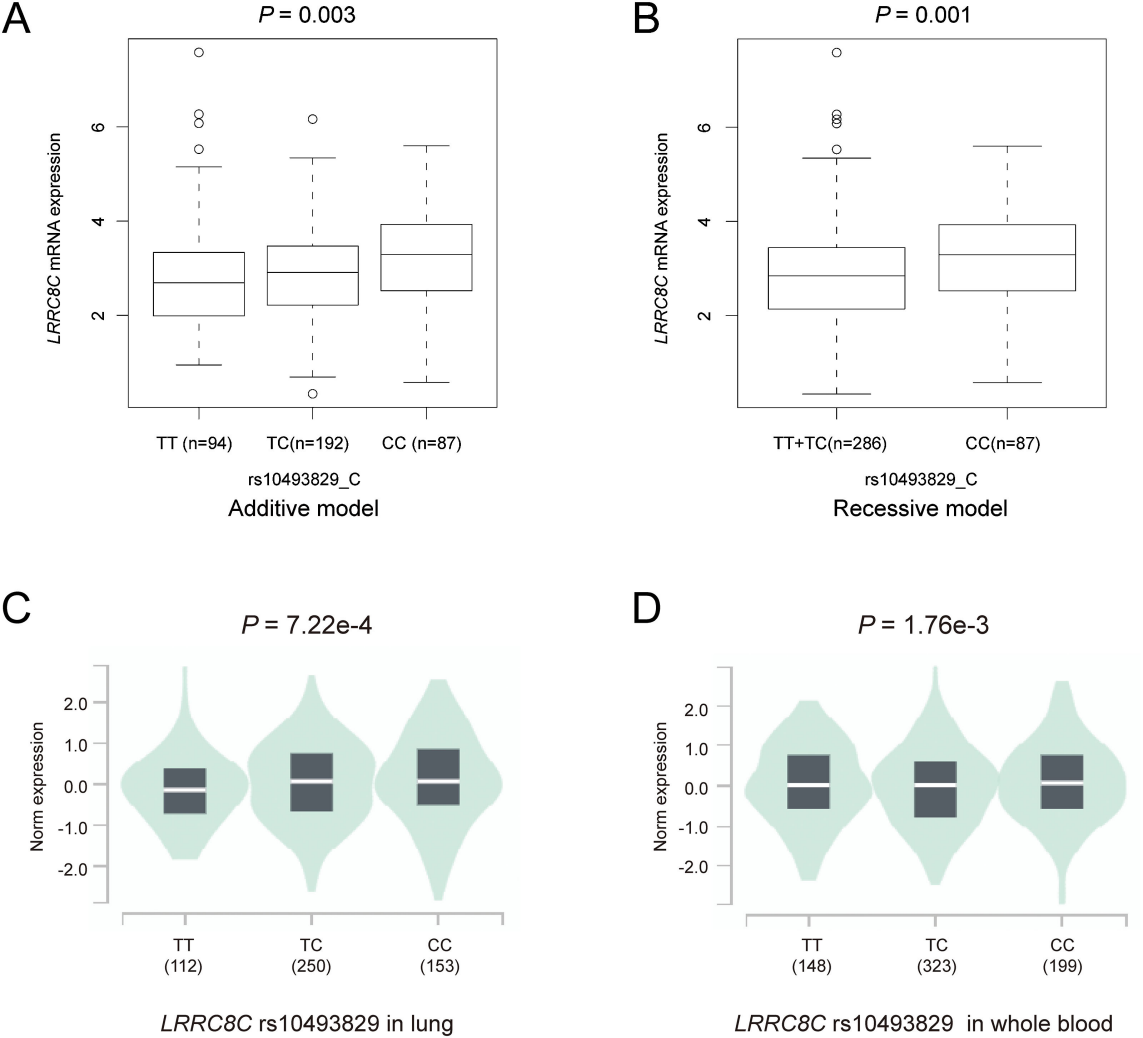
The results of eQTL analyses. The *LRRC8C* rs10493829 C allele was associated with high mRNA expression levels of *LRRC8C* in **(A)** additive model and **(B)** recessive model in normal lymphoblastoid cells, **(C)** normal lung tissues and **(D)** whole blood samples in the GTEx project.

To explore potential mechanisms underlying the associations of three independent SNPs with NSCLC survival, we performed eQTL analyses to investigate the correlations between these three independent SNPs and their corresponding mRNA expression levels. First, with RNA-Seq data of the lymphoblastoid cell lines from 373 European descendants in the 1000 Genomes Project, the *LRRC8C* rs10493829 C allele was significantly correlated with increased expression levels of *LRRC8C* mRNA in additive (*P* = 0.003, **Figure 6A**) and recessive models (*P* = 0.001, **Figure 6B**), but not in the dominant model (**Supplementary Figure 5A**). The *OAS2* rs2239193 G allele and *CCL25* rs3136651 A allele showed no correlation with their mRNA expression levels in additive, dominant, and recessive models (**Supplementary Figures 5B–G**). Then, using data from the GTEx project, we found that the *LRRC8C* rs10493829 C allele was significantly correlated with high mRNA expression levels of *LRRC8C* in normal lung tissues (*P* = 7.22e-4, **Figure 6C**) and whole blood samples (*P* = 1.76e-3, **Figure 6D**). The *OAS2* rs2239193 G allele showed no correlation with *OAS2* mRNA expression levels in normal lung tissues and whole blood (**Supplementary Figures 5H, I**). However, there was no data for *CCL25* rs3136651 A allele in the GTEx project. These data indicated that the *LRRC8C* rs10493829 C allele might regulate the mRNA expression levels of its corresponding gene.

### 3.7 The result of mRNA expression levels and survival

**Figure 7:**
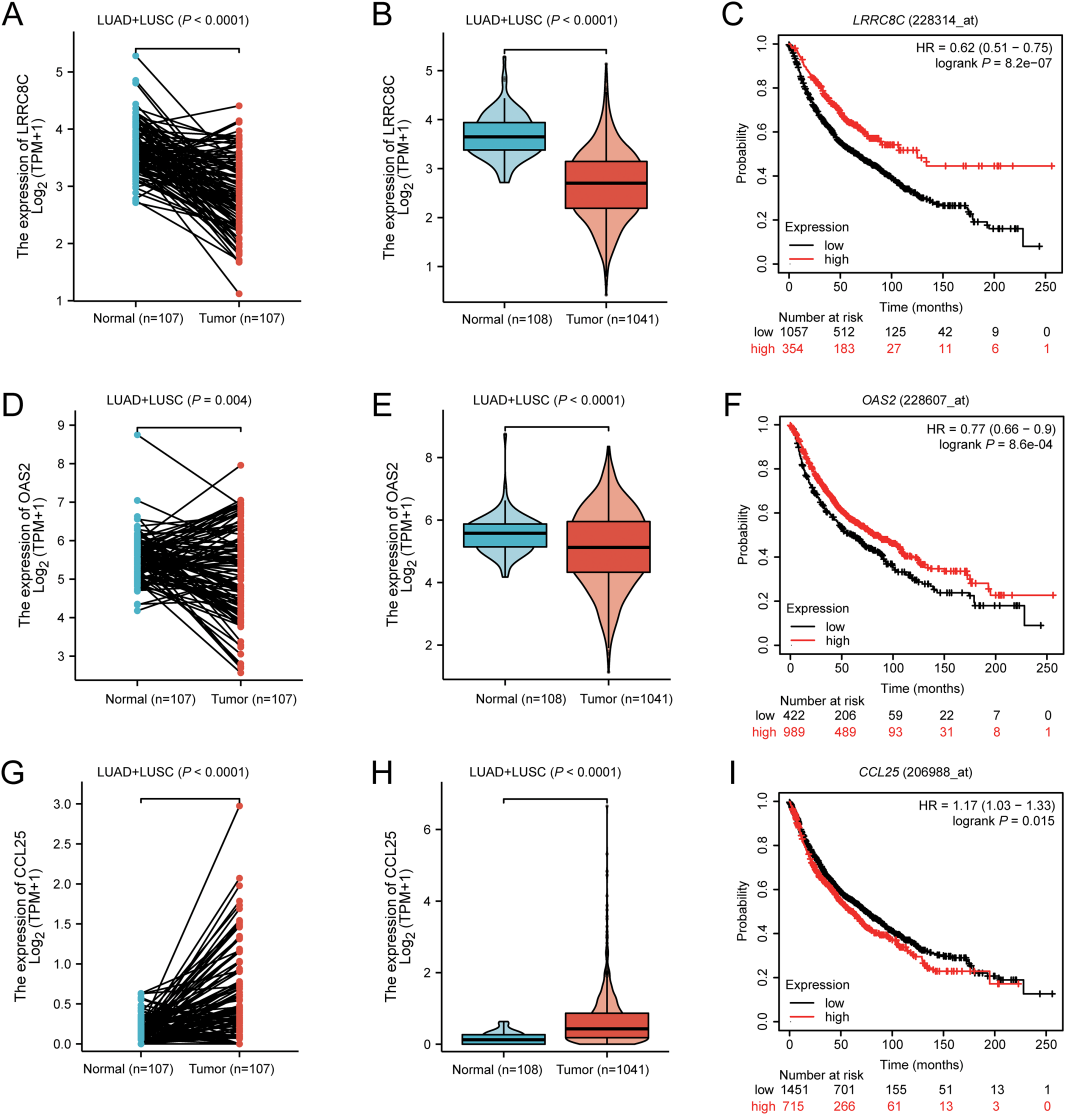
The result of mRNA expression levels from XIANTAO and survival from Kaplan-Meier Plotter database for *LRRC8C*, *OAS2*, and *CCL25*. *LRRC8C* mRNA was down-regulated in combined LUAD+LUSC with **(A)** paired and **(B)** unpaired tests, **(C)** high *LRRC8C* mRNA expression levels were associated with a better NSCLC survival; *OAS2* mRNA was down-regulated in combined LUAD+LUSC with **(D)** paired and **(E)** unpaired tests, **(F)** high *OAS2* mRNA expression levels were associated with a better NSCLC survival; *CCL25* mRNA was up-regulated in combined LUAD+LUSC with **(G)** paired and **(H)** unpaired tests, **(I)** high *CCL25* mRNA expression levels were associated with a poor NSCLC survival.

To explore the mRNA expression levels of *LRRC8C*, *OAS2*, and *CCL25* with paired and unpaired tests, we input these three genes in the module “paired samples” and “disease and non-disease” of the XIANTAO online tool. Paired *t*-tests suggested that compared with normal lung tissue, *LRRC8C* mRNA was significantly down-regulated in combined lung squamous cell carcinoma (LUSC) + lung adenocarcinoma (LUAD) (**Figure 7A**), LUSC, and LUAD (all *P <* 0.0001) (**Supplementary Figures 6A, B**). Similar results were also observed with unpaired tests for combined LUSC + LUAD (**Figure 7B**), LUSC, and LUAD (**Supplementary Figures 6C, D**). Furthermore, survival analysis from the Kaplan-Meier Plotter database suggested that high *LRRC8C* mRNA expression levels were associated with a better NSCLC survival (HR = 0.62, 95% CI: 0.51-0.75, log-rank *P <* 0.0001) (**Figure 7C**). Paired tests suggested that *OAS2* mRNA expression levels were down-regulated in combined LUSC + LUAD (*P* = 0.004) (**Figure 7D**), LUSC (*P* = 0.026), but not in LUAD (*P* = 0.066) (**Supplementary Figures 6E, F**), and the unpaired tests indicated that mRNA expression levels of *OAS2* were lower in LUSC + LUAD (*P* = 4.6e-05) (**Figure 7E**), LUSC (*P* = 0.003), and LUAD (*P* = 0.006) (**Supplementary Figures 6G, H**). Moreover, high *OAS2* mRNA expression levels were associated with favorable NSCLC survival (HR = 0.77, 95% CI: 0.66-0.90, log-rank *P* = 8.6e-04) (**Figure 7F**). Furthermore, paired and unpaired tests indicated that the mRNA expression levels of *CCL25* were up-regulated in combined LUSC + LUAD, LUSC, and LUAD (**Figures 7G, H**, **Supplementary Figures 6–L**). High mRNA expression levels of *CCL25* were associated with a poor NSCLC OS (HR = 1.17, 95% CI: 1.03-1.33, log-rank *P* = 0.015) (**Figure 7I**). Taken together, these findings suggested that *LRRC8C* and *OAS2* might act as suppressor genes, while *CCL25* might function as an oncogene.

## 4 Discussion

In the present study, we assessed the associations between 52,103 SNPs in the 672 T cell exhaustion-related genes and NSCLC survival by using available NSCLC genotyping data from two public GWAS datasets and clinical information from both the PLCO trial and the HLCS study. We identified three SNPs (i.e., the *LRRC8C* rs10493829 T>C, *OAS2* rs2239193 A>G, and *CCL25* rs3136651 T>A) that were independently associated with the survival of NSCLC patients. Additionally, an increased NUG score was associated with poorer NSCLC OS and DSS. Furthermore, the addition of these three SNPs to the predictive model significantly improved 5-year DSS, suggesting that these three independent SNPs may be predictors for NSCLC survival. In the functional SNP-mRNA analysis, we also found that *LRRC8C* rs10493829 C allele was associated with significantly higher *LRRC8C* mRNA expression levels. Moreover, high *LRRC8C* and *OAS2* mRNA expression levels were associated with a better NSCLC survival, while high *CCL25* mRNA expression levels were associated with a poorer NSCLC survival. These data indicated that *LRRC8C* rs10493829 C allele could modulate the mRNA expression levels of *LRRC8C* to influence NSCLC survival, which provided some evidence for biological plausibility of the observed SNP-survival associations, particularly for the *LRRC8C* rs10493829 T>C SNP.

Recent advances in immunotherapy have dramatically improved the prognosis of NSCLC patients; however, the acquired resistance limits the percentage of patients with durable therapeutic responses (39). T cell exhaustion, a hypofunctional state of T cells resulting from a prolonged exposure to antigenic stimulation, is a key hallmark of the immunosuppressive TME status and mechanism of the acquired resistance to immunotherapy (40, 41). However, there was no reported role of genetic variants in the T cell exhaustion-related genes on survival of NSCLC patients. For the first time, in the present study, we identified three SNPs in T cell exhaustion-related genes, which collectively predicted NSCLC survival.

*LRRC8C*, the leucine-rich repeat containing 8 volume-regulated anion channel (VRAC) subunit C, is located on chromosome 1 and composed of 803 amino acids. Previous studies of *LRRC8C* have focused on the immune system. As an essential component for VRAC, *LRRC8C* mediates the transport of 2’3cGAMP and activates STING and P53 to inhibit the enhanced T cell function, further regulating T cell proliferation and survival (42). On the contrary, the deletion of Lrrc8c enhanced the CD4^+^ and CD8^+^ T cell function by down-regulating p53 signaling (42, 43). These discoveries added to the evidence that *LRRC8C*-STING-p53 signaling axis may act as a new inhibitory pathway that controls the function and adaptive immunity of T cells.

However, what is not yet known is the role of *LRRC8C* in NSCLC survival. To the best of our knowledge, the present study is the first to to have identified the associations between genetic variants of *LRRC8C* and NSCLC survival. Notably, *LRRC8C* rs10493829 T>C showed a significant protective effect on the survival of NSCLC patients and was associated with elevated mRNA expression levels in normal lymphoblastoid cells, lung tissue, and whole blood. Furthermore, *LRRC8C* mRNA expression levels were upregulated in normal tissues and associated with favorable NSCLC survival. These findings imply that *LRRC8C* may act as a suppressor gene and that the *LRRC8C* rs10493829 C allele may regulate the mRNA expression levels of its corresponding gene to influence prognosis in NSCLC.

*OAS2*, 2’-5’-Oligoadenylate Synthetase 2, is located on chromosome 12 and composed of 719 amino acids. Previous studies have suggested that *OAS2* regulates multiple cellular processes, including cell proliferation, invasion, and autophagy. For example, it was reported that *OAS2* suppressed cell proliferation and invasion and promoted autophagy in colorectal cancer (44). Moreover, *OAS2* overexpression was found to be significantly associated with a favorable prognosis in various cancers (44, 45). As for NSCLC, *OAS2* was significantly down-regulated in human gefitinib-resistant tissues, while up-regulation of *OAS2* reversed the resistance in gefitinib-resistant cell lines (46). In the present study, we found that the *OAS2* rs2239193 G allele was associated with NSCLC survival. In addition, *OAS2* mRNA expression levels were increased in normal lung tissues than NSCLC tissues, and up-regulation of *OAS2* was associated with favorable NSCLC survival. Consistent with previous studies, these findings also suggested that *OAS2* might function as a potential suppressor gene in NSCLC. However, we did not find an association between *OAS2* rs2239193 G allele and mRNA expression levels of *OAS2*. Taken together, additional experiments should be designed to explore the potential mechanisms underlying the observed associations.

*CCL25*, C-C Motif Chemokine Ligand 25, is located on chromosome 19 and composed of 150 amino acids. CCL25 is the natural ligand for C-C motif chemokine receptor 9 (CCR9). Data from previous studies have established that CCR9/*CCL25* interaction promote tumor proliferation, invasion, anti-apoptosis, and migration in a variety of malignant tumors (47–49). In NSCLC, the CCR9/*CCL25* interaction induced tumorigenesis and inhibited apoptosis of tumor cells by activating the PI3K/Akt signaling pathway (50). Similarly, another study demonstrated that *CCL25* enhanced the phenotype of migration and invasion in NSCLC lines and that NSCLC patients with lower *CCL25* expression had a better OS (51). Moreover, the CCR9/CCL25 chemokine axis plays an important role in shaping TME by attracting immune cells in the tumor, leading TME toward an immunosuppressive state (52). In the present study, we found that the *CCL25* rs3136651 A allele had a significant protective effect on NSCLC survival and that *CCL25* mRNA levels were markedly up-regulated in NSCLC tissues and associated with a reduced survival. These results were consistent with the above-mentioned previous studies, suggesting that *CCL25* may function as an oncogene in NSCLC; however, additional functional studies should be designed to investigate the underlying molecular mechanisms.

There are several limitations in the present study. Firstly, although the evidence showing that genetic variants in T cell exhaustion-related genes are associated with NSCLC survival, the molecular mechanisms underlying the observed associations are still uncertain. Further experiments *in vitro* and *in vivo* should be designed to investigate the potential mechanisms. Secondly, because the two available GWAS datasets were of European descendants, our results may not be generalized to other ethnic populations. Thirdly, the detailed genotype and clinical outcome data were not available from HLCS for us to replicate the results of the same combined and stratified analyses performed with the PLCO data only.

In conclusion, in the present study, we identified that three independent SNPs were associated with NSCLC survival in both the PLCO trial and the HLCS study. We also found that *LRRC8C* rs10493829 C allele affected NSCLC survival possibly by regulating the targeted mRNA expression. Our results indicate that these three SNPs in the T cell exhaustion-related genes may be potential biomarkers for NSCLC survival.

## Data Availability

The datasets presented in this study can be found in online repositories. The names of the repository/repositories and accession number(s) can be found below: https://www.ncbi.nlm.nih.gov/gap/, phs000093.v2.P2; https://www.ncbi.nlm.nih.gov/snp/, phs000336.v1.p1.
