# Supplementary material for: Genetic variants of LRRC8C, OAS2, and CCL25 in the T cell exhaustion-related genes are associated with non-small cell lung cancer survival
Source: Front Immunol. 2024 Oct 2;15:1455927. doi: 10.3389/fimmu.2024.1455927 (PMC11479925; doi:10.3389/fimmu.2024.1455927)
Supplement: Supplementary file 1 [file DataSheet1.doc]

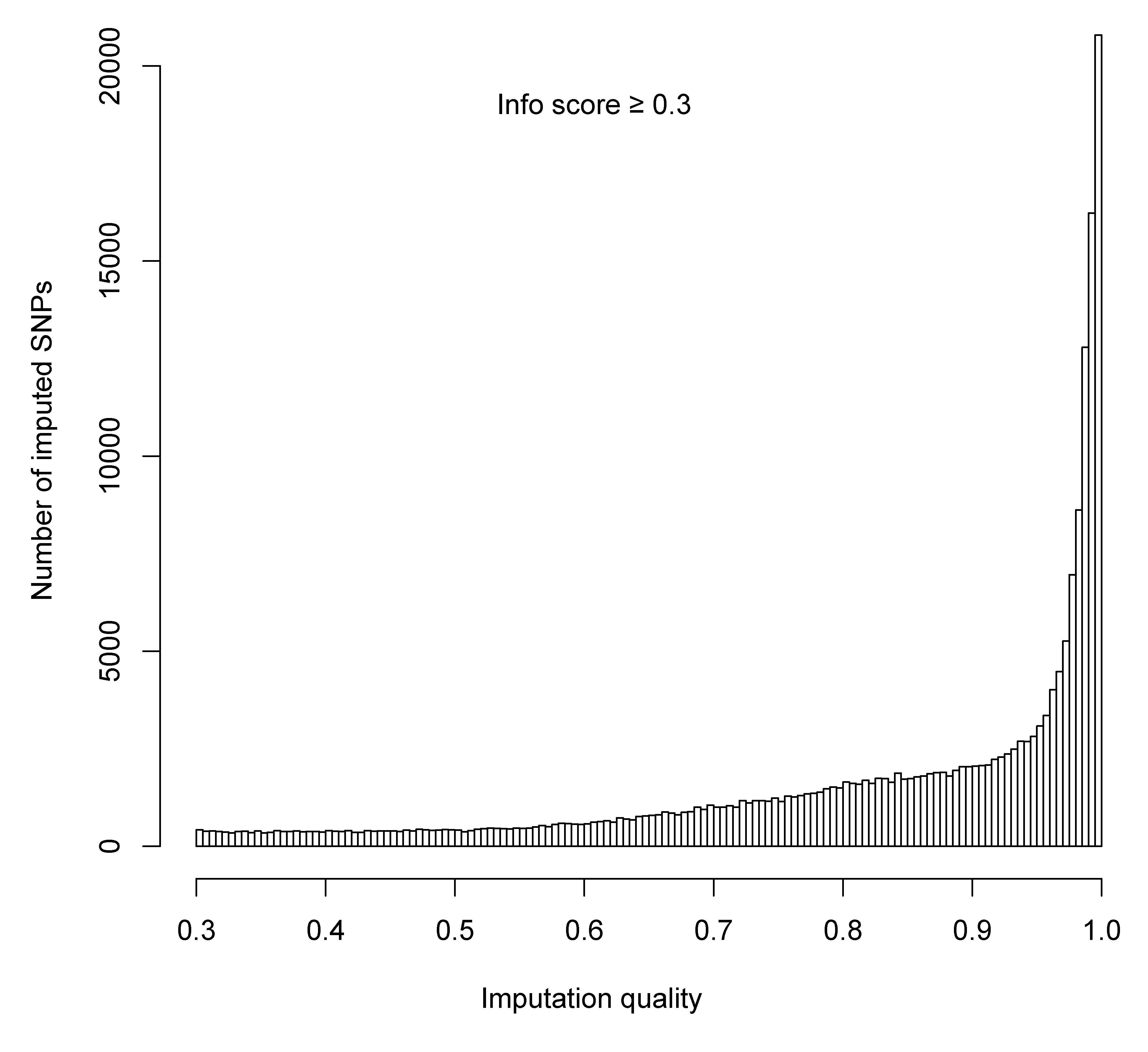


**Supplementary Figure 1:** The distribution of the imputation information score of the present study.


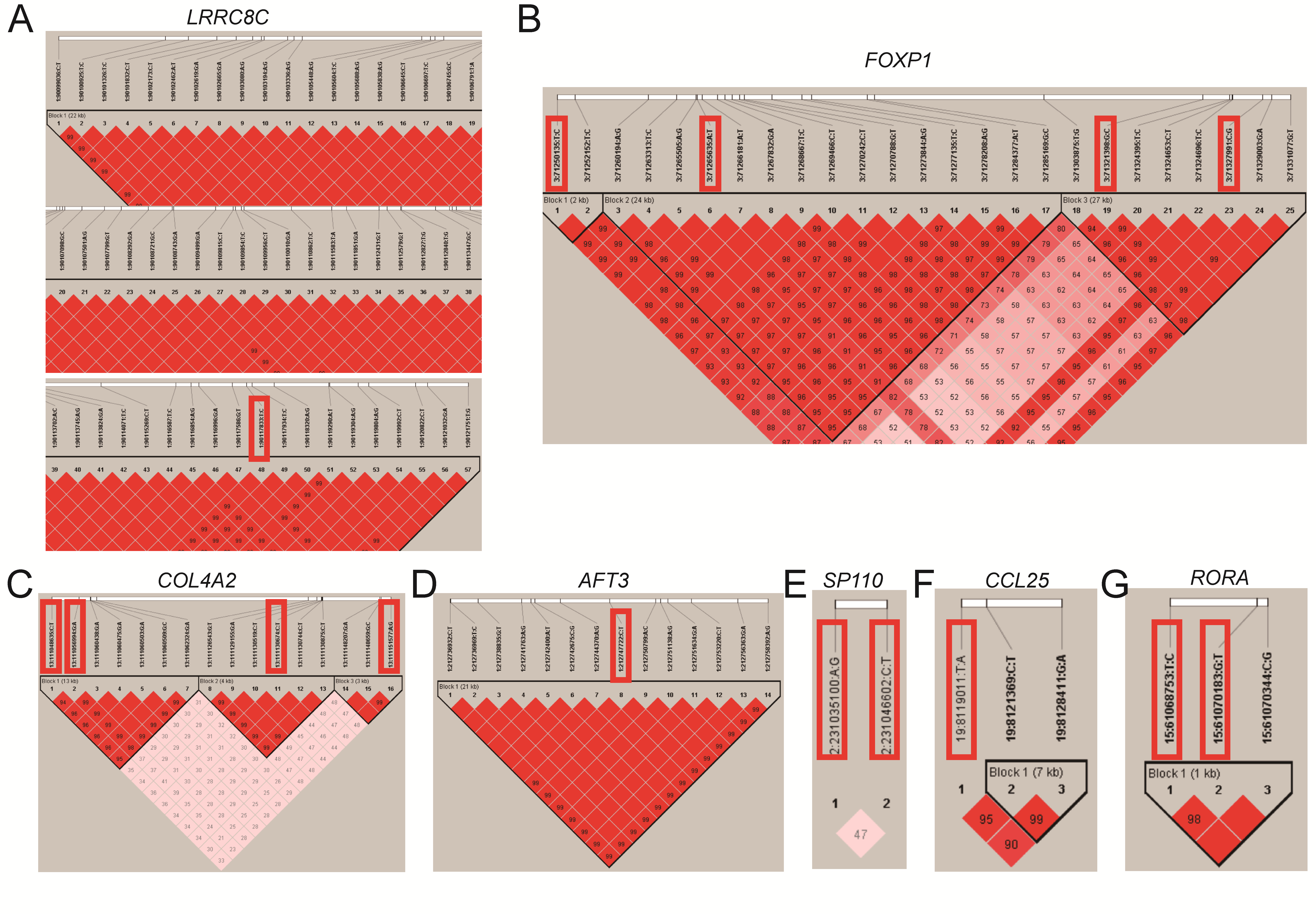


**Supplementary Figure 2:** Linkage disequilibrium (LD) analysis for 15 significant SNPs located in 7 genes. (**A**) 57 SNPs in *LRRC8C*, (**B**) 25 SNPs in *FOXP1*, (**C**) 16 SNPs in *COL4A2*, (**D**) 14 SNPs in *ATF3*, (**E**) 2 SNPs in *SP110*, (**F**) 3 SNPs in *CCL25*, (**G**) 3 SNPs in *RORA*.

Abbreviations: SNPs, single nucleotide polymorphism.


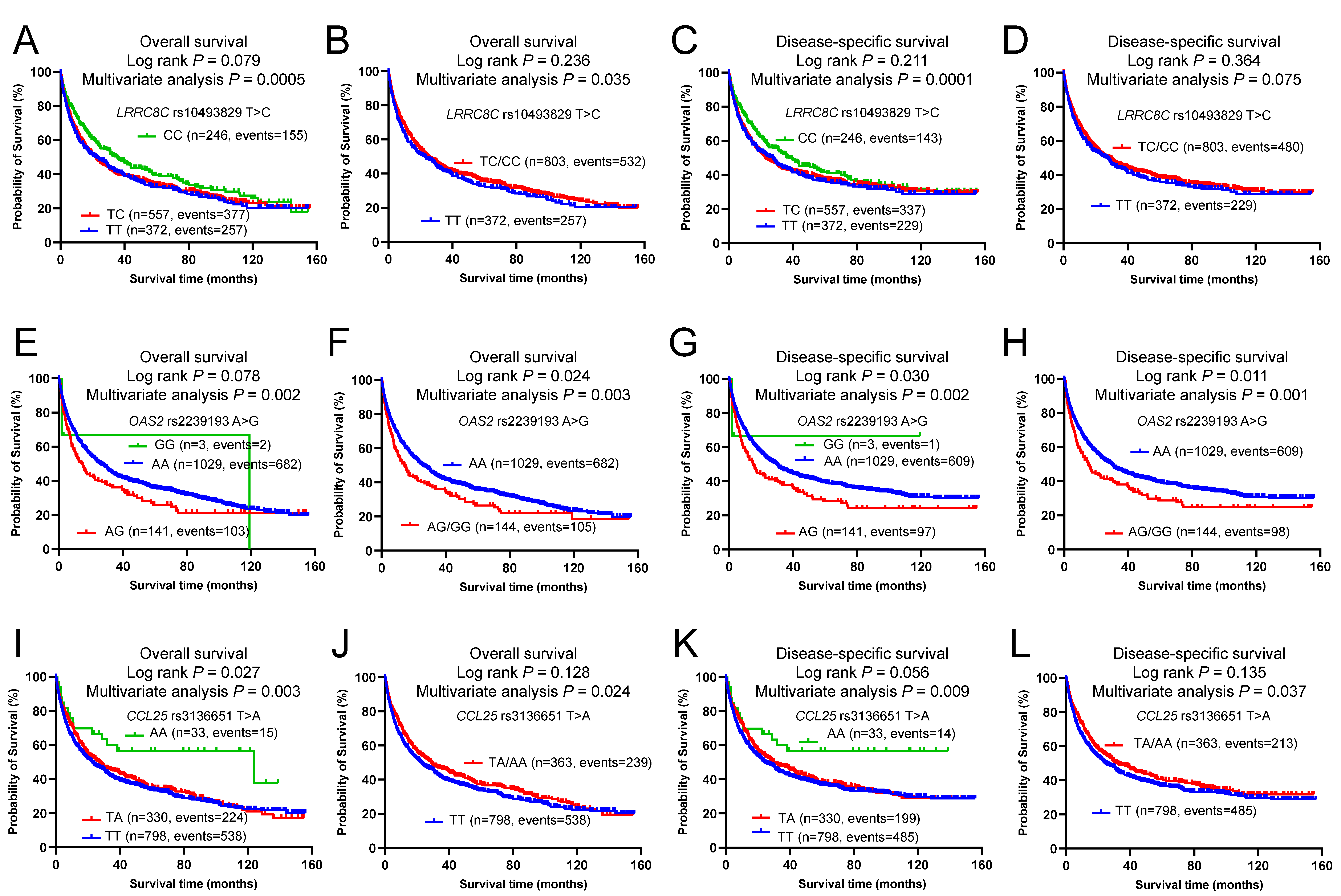


**Supplementary Figure 3**. Prediction of OS and DSS survival with genotypes of *LRRC8C* rs10493829, *OAS2* rs2239193 and *CCL25* rs3136651 in the PLCO dataset. Kaplan-Meier survival curves of *LRRC8C* rs10493829 for OS with (**A**) additive model, (**B**) dominant model; for DSS with (**C**) additive model, (**D**) dominant model; *OAS2* rs2239193 for OS with (**E**) additive model; (**F**) dominant model; for DSS with (**G**) additive model; (**H**) dominant model; *CCL25* rs3136651 for OS with (**I**) additive model; (**J**) dominant model; for DSS with (**K**) additive model; (**L**) dominant model.


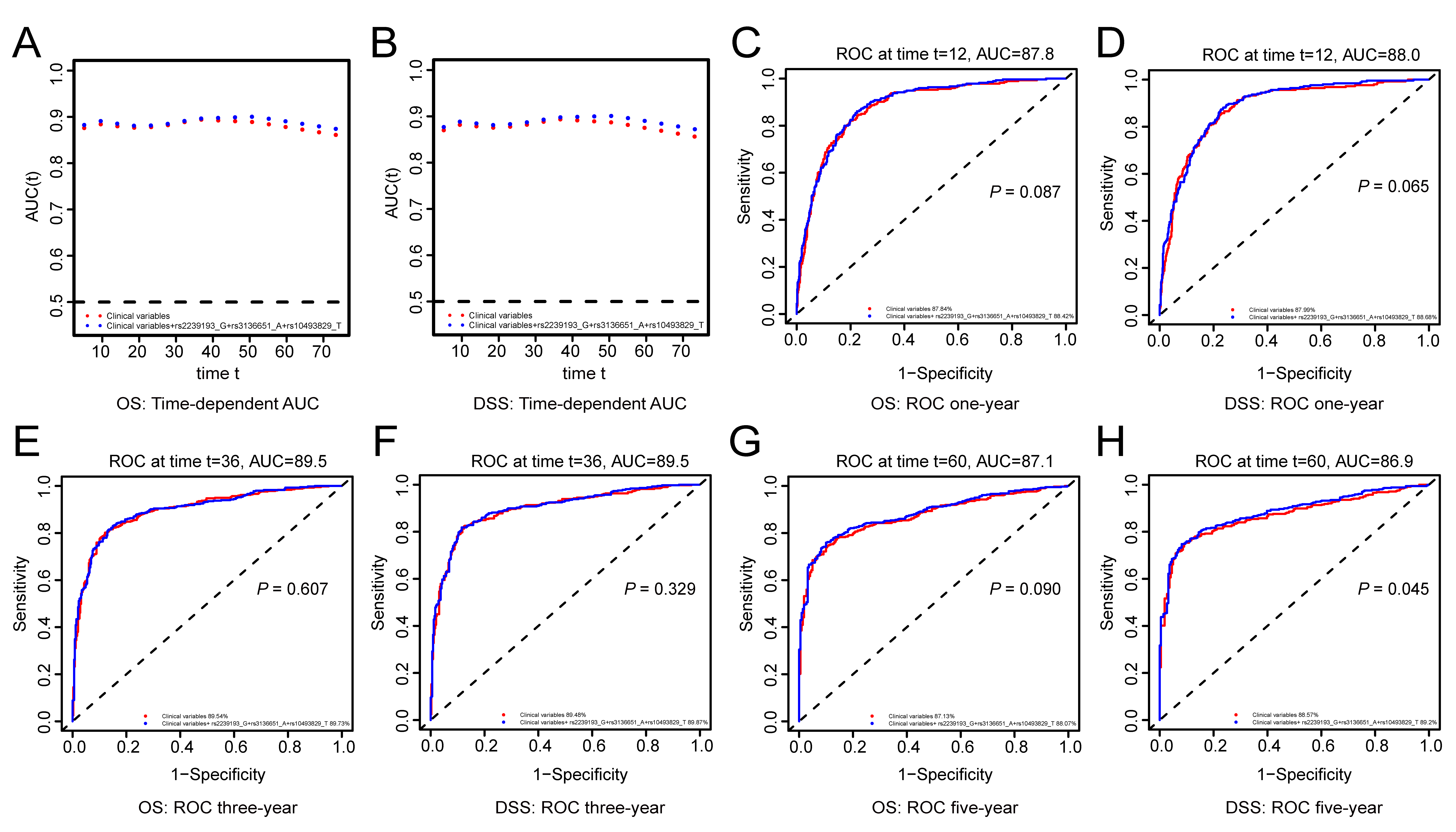


**Supplementary Figure 4.** NSCLC survival prediction with three SNPs by AUC and ROC curve at the 12^th^ ,36^th^, and 60^th^ month. Time-dependent AUC analysis of (**A**) OS and (**B**) DSS based on age, sex, smoking condition, histology, tumor stage, chemotherapy, surgery, principal component and the two SNPs; The ROC curve evaluation at 12^th^ month for (**C**) OS and (**D**) DSS, 36^th^ month for (**E**) OS and (**F**) DSS, 60^th^ month for (**G**) OS and (**H**) DSS.


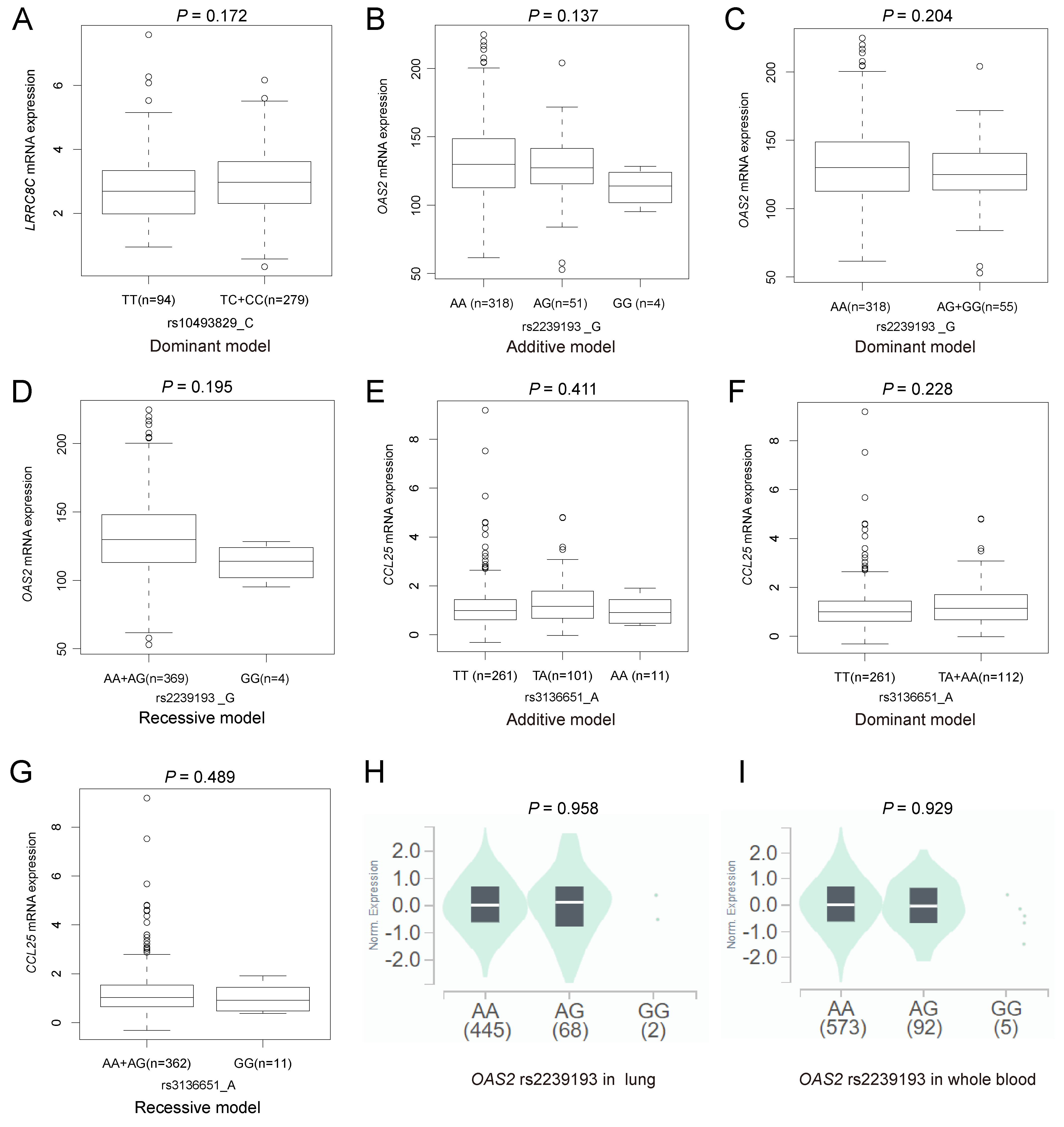


**Supplementary Figure 5.** Correlation between three genotypes and their corresponding mRNA expression levels.

The eQTL from 1000 Genomes project for *LRRC8C* rs10493829 in (**A**) dominant model; for *OAS2* rs2239193 in (**B**) additive model, (**C**) dominant model, and (**D**) recessive model; for *CCL25* rs3136651 in (**E**) additive model, (**F**) dominant model, and (**G**) recessive model. The eQTL from GTEx project for *OAS2* rs2239193 in (**H**) normal lung tissue and (**I**) whole blood samples.


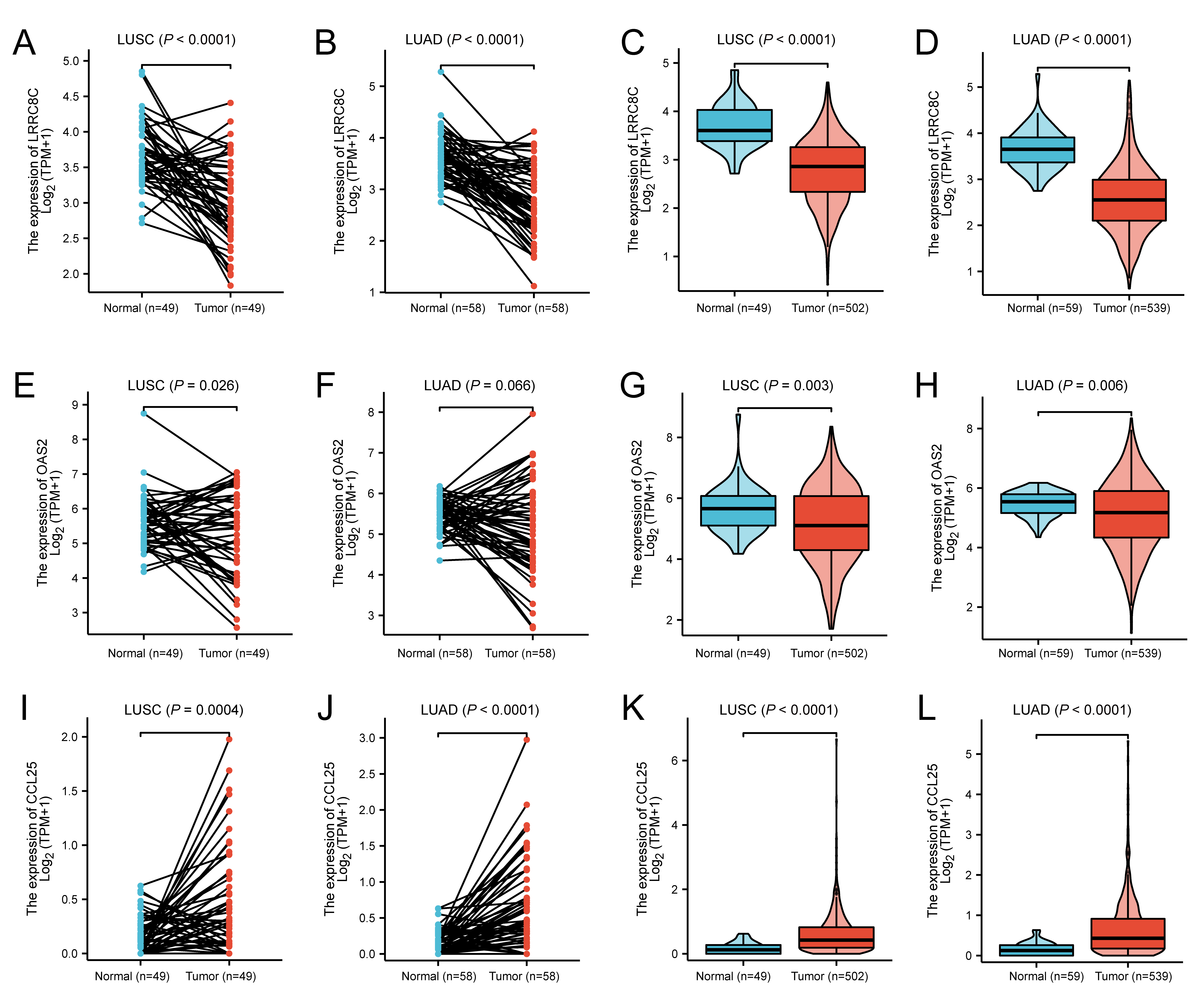


**Supplementary Figure 6**. Paired and non-paired mRNA expression analysis of *IRRC8C*, *OAS2*, and *CCL25.*

Paired and unpaired tests suggested that *IRRC8C* mRNA was significantly downregulated in (**A**-**D**) LUSC and LUAD; Paired t-test suggested that *OAS2* mRNA expression level was downregulated in (**E**) LUSC, but not in (**F**) LUAD; Unpaired tests suggested that *OAS2* was downregulated in (**G**) LUSC and (**H**) LUAD; Paired and unpaired tests suggested that *CCL25* mRNA was upregulated in (**I**-**L**) LUSC and LUAD.
