# Supplementary material for: Genetic variants of LRRC8C, OAS2, and CCL25 in the T cell exhaustion-related genes are associated with non-small cell lung cancer survival
Source: Front Immunol. 2024 Oct 2;15:1455927. doi: 10.3389/fimmu.2024.1455927 (PMC11479925; doi:10.3389/fimmu.2024.1455927)
Supplement: Supplementary file 2 [file Table1.docx]

| **Supplementary Table 1.** Comparison of characteristics between the PLCO trial and the HLCS study | | | | | | |
| --- | --- | --- | --- | --- | --- | --- |
| **Characteristics** | **PLCO** | |  | **HLCS** | | ***P* *** |
|  | **Frequency** | **Deaths (%)** |  | **Frequency** | **Deaths (%)** |  |
| Total | 1,185 | 798 (67.3) |  | 984 | 665 (67.5) |  |
| Median overall survival (months) | 23.8 |  |  | 39.9 |  |  |
| Age |  |  |  |  |  |  |
| ≤71 | 636 | 400 (62.9) |  | 654 | 428 (65.4) | <0.0001 |
| >71 | 549 | 398 (72.5) |  | 330 | 237 (71.8) |  |
| Sex |  |  |  |  |  |  |
| Male | 698 | 507 (72.6) |  | 507 | 379 (74.7) | 0.0006 |
| Female | 487 | 291 (59.8) |  | 477 | 286 (59.9) |  |
| Smoking status |  |  |  |  |  |  |
| Never | 115 | 63 (54.8) |  | 92 | 52 (56.5) | 0.166 |
| Current | 423 | 272 (64.3) |  | 390 | 266 (68.2) |  |
| Former | 647 | 463 (71.6) |  | 502 | 347 (69.1) |  |
| Histology |  |  |  |  |  |  |
| Adenocarcinoma | 577 | 348 (60.3) |  | 597 | 378 (63.3) | <0.0001 |
| Squamous cell carcinoma | 285 | 192 (67.4) |  | 216 | 156 (72.2) |  |
| Others | 323 | 258 (79.9) |  | 171 | 131 (76.6) |  |
| Stage |  |  |  |  |  |  |
| I - IIIA | 655 | 315 (48.1) |  | 606 | 352 (58.0) | 0.003 |
| IIIB - IV | 528 | 482 (91.3) |  | 377 | 313 (83.0) |  |
| *Missing* | 2 |  |  | -- |  |  |
| Abbreviations: PLCO, the Prostate, Lung, Colorectal and Ovarian Cancer Screening Trial; HLCS, Harvard Lung Cancer Susceptibility Study.  * Chi-square test for the comparison of characteristics between the PLCO trial and the HLCS study for each clinical variable. | | | | | | |

| **Supplementary Table 2.** List of 672 TEX-related genes used in the discovery analysis (from reference 22) | | | | |
| --- | --- | --- | --- | --- |
| **Dataset** | **Selected genes ^a^** | | **Number**  **of genes** |  |
| TEX-related  genes | *ABCA1, ABCB1, ACE, ACE2, ACKR3, ADAM19, ADAR, AGER, AGTR1, AHCY, AHNAK, AHR, ALAS2, ALCAM, ALDOA, ALOX5AP, AMACR, ANXA4, APEX1, APLP1, APOL6, AR, AREG, ARID5B, ARL4A, ARL4C, ARNT, ARNT2, ASS1, ATF3, ATP2B1, AUTS2, B2M, B3GAT1, B4GALT1, B4GALT5, BACH2, BANK1, BATF, BATF2, BATF3, BAX, BCL2, BCL2A1, BCL2L1, BCL3, BCL6, BHLHE40, BID, BIN2, BIRC2, BIRC3, BMP2, BMPR2, BNIP3, BPGM, BST2, BTG1, BTG2, BTG3, C1orf162, C1orf21, C1R, C1S, CA12, CA2, CA9, CAPG, APN3, CASP1, CASP3, CASP4, CASP7, CASP8, CCL1, CCL10, CCL11, CCL12, CCL13, CCL14, CCL15, CCL16, CCL17, CCL18, CCL19, CCL2, CCL20, CCL21, CCL22, CCL23, CCL24, CCL25, CCL26, CCL27, CCL28, CCL3, CCL4, CCL5, CCL6, CCL7, CCL8, CCL9, CCN1, CCND1, CCND2, CCND3, CCNE1, CCNL1, CCR4, CCR7, CCRL2, CD244, CD274, CD300A, CD38, CD40, CD44, CD48, CD69, CD74, CD79B, CD80, CD81, CD83, CD86, CDC42SE2, CDC6, CDCP1, CDH1, CDKN1A, CDKN1B, CDKN1C, CEBPB, CEBPD, CFB, CFH, CFLAR, CIITA, CISH, CKAP4, CLCF1, CMKLR1, CMPK2, CMTR1, COCH, COL4A2, COL6A1, CSF1, CSF2, CSF2RB, CST7, CTGF, CTLA4, CTSW, CTSZ, CX3CR1, CXCL1, CXCL10, CXCL11, CXCL12, CXCL2, CXCL3, CXCL6, CXCL9, CXCR3, CXCR4, CYFIP1, DAB2, DCPS, DDX58, DDX60, DENND5A, DHRS3, DHX58, DNAJB4, DRAM1, DRC1, DUSP1, DUSP2, DUSP4, DUSP5, E2F3, ECM1, EDN1, EDNRB, EEF1AKMT1, EFHD2, EFNA1, EGLN1, EGLN3, EGR1, EGR2, EGR3, EHD1, EIF1, EIF2AK2, EIF4E3, EMP1, EMP3, ENO1, ENO3, ENPP1, EOMES, EPO, EPSTI1, ERCC2, ETFBKMT, ETS2, ETV4, F2RL1, F2RL2, F3, FAH, FAM126B, FAM162A, FAM65B, FAS, FCGR1A, FCGR3A, FCRL6, FGF2, FGFBP2, FGL2, FGR, FJX1, FLT3LG, FOS, FOSB, FOSL1, FOSL2, FOXO1, FOXP1, FPR1, FURIN, FUT4, G0S2, GABARAPL1, GADD45A, GADD45B, GALM, GAPDH, GATA1, GBP4, GBP6, GCH1, GCK, GEM, GFPT2, GLIPR2, GNLY, GPR18, GPR183, GPR65, GPR83, GPX4, GSTO1, GUCY1B1, GZMA, GZMB, HAVCR2, HBEGF, HELZ2, HERC6, HES1, HIF1A, HIPK2, HK2, HLA-A, HLA-B, HLA-DMA, HLA-DQA1, HLA-DRB1, HLA-G, HOPX, HUWE1, HZMH, ICAM1, ICOS, ICOSLG, ID2, IDO1, IER2, IER3, IER5, IFI27, IFI30, IFI35, IFI44, IFI44L, IFIH1, IFIT1, IFIT2, IFIT3, IFITM2, IFITM3, IFNAR2, IFNG, IFNGR1, IFNGR2, IGF1R, IGF2R, IKZF2, IKZF4, IL10, IL10RA, IL12A, IL12B, IL13, IL15, IL15RA, IL17A, IL18, IL18BP, IL18R1, IL1A, IL1B, IL1R2, IL1RL1, IL23A, IL2RA, IL2RB, IL3RA, IL4R, IL5, IL6, IL6ST, IL7, IL7R, INHBA, IRF1, IRF2, IRF4, IRF5, IRF6, IRF7, IRF8, IRF9, IRS2, ISG15, ISG20, ISOC1, ITGA6, ITGAE, ITGAV, ITGB2, ITGB7, ITIH5, JAG1, JAK2, JUN, JUNB, KDM6B, KLF10, KLF2, KLF4, KLF6, KLF9, KLK3, KLRD1, KLRF1, KLRG1, KLRK1, KLRKB1, KYNU, LAG3, LAMB3, LAP3, LATS2, LCLAT1, LCP2, LDHA, LDLR, LEP, LGALS3BP, LIF, LITAF, LOX, LRIG1, LRRC8C, LTB, LY6E, LYSMD2, MAFF, MAP2K3, MAP3K8, MAP6, MAPKAPK2, MARCHF1, MARCKS, MCL1, MET, METTL7B, MICA, MIF, MMP2, MSC, MT2A, MTHFD2, MUC1, MUC3A, MVP, MX1, MX2, MXD1, MYC, MYD88, MYO1C, MYO1E, NAMPT, NCOA3, NCS1, NDRG1, NFAT5, NFE2L2, NFIL3, NFKB1, NFKB2, NFKBIA, NFKBIE, NFKBIZ, NINJ1, NKG7, NLRC5, NMI, NMU, NOD1, NOP2, NR3C1, NR4A1, NR4A2, NR4A3, NRP1, NT5E, NUP93, OAS2, OAS3, OASL, ODC1, OGFR, OLR1, P2RX4, P2RY14, P4HA1, PANX1, PARP12, PARP14, PDCD1, PDCD2L, PDE4B, PDLIM5, PELI1, PENK, PER1, PFKFB3, PFKFB4, PFKP, PGK1, PHLDA1, PHLDA2, PHTF2, PIK3CA, PIM1, PLA2G4A, PLAC8, PLAGL1, PLAU, PLAUR, PLEC, PLEK, PLIN2, PLK2, PLPP1, PLPP3, PLSCR1, PMEPA1, PML, PNP, PNPT1, PNRC1, POU2F1, PPARA, PPP1R15A, PPP1R3C, PRAF2, PRDM1, PRF1, PRKCH, PRNP, PSMA2, PSMA3, PSMB10, PSMB2, PSMB8, PSMB9, PSME1, PSME2, PTCH1, PTGER2, PTGER4, PTGES, PTGS2, PTH1R, PTPN1, PTPN2, PTPN6, PTPRE, PTRH2, PTX3, PUS1, RABGAP1L, RAPGEF6, RBCK1, RCAN1, RECK, REL, RELA, RELB, RGS16, RHOB, RHOH, RIPK1, RIPK2, RNF19B, RNF213, RNF31, RNH1, RORA, RRAGD, RSAD2, RTP4, S100A1, S1PR1, S1PR5, SAMD3, SAMD9L, SAMHD1, SAT1, SCN9A, SDC4, SDHB, SECTM1, SELL, SELP, SERPINB2, SERPINB6, SERPINB8, SERPINC1, SERPINE1, SERPING1, SETD2, SGK1, SH3BGRL2, SHE, SIK1, SLAMF6, SLAMF7, SLC16A6, SLC1A5, SLC25A28, SLC29A1, SLC29A2, SLC2A1, SLC2A3, SLC2A6, SLC39A8, SMAD3, SMPDL3A, SNN, SNX14, SNX9, SOCS1, SOCS2, SOCS3, SOD2, SORL1, SP110, SPARC, SPHK1, SPON2, SPP1, SPPL2A, SPRED2, SPRY4, SPSB1, SQSTM1, SRI, SSPN, ST3GAL4, ST3GAL5, ST8SIA4, STAT1, STAT2, STAT3, STAT4, STAT5A, STK38, SWAP70, SYNGR2, SYT11, TANK, TAP1, TAPBP, TBX21, TCF4, TCF7, TDRD7, TERT, TFRC, TGFB3, TGFBR3, TGIF1, TGM2, TIAM1, TIGIT, TIMP2, TIPARP, TLR2, TLR6, TLR7, TNC, TNF, TNFAIP2, TNFAIP3, TNFAIP6, TNFAIP8, TNFRSF18, TNFRSF1B, TNFRSF21, TNFRSF4, TNFRSF8, TNFRSF9, TNFSF10, TNFSF11, TNFSF9, TNIP1, TNIP2, TOR1B, TOX, TP53, TRAF1, TRAFD1, TRDC, TRIB1, TRIM14, TRIM21, TRIM25, TRIM26, TRIP10, TSC22D1, TTC39B, TUBB2A, TUBB3, TWIST1, TWIST2, TWSG1, TXNIP, TYROBP, UBE2L6, UCK2, UMPS, UPP1, USP18, VAMP5, VAMP8, VCAM1, VEGFA, VEGFB, VHL, VIM, WARS1, WASF3, WLS, XAF1, XBP1, XCL1, XPC, YRDC, ZBP1, ZBTB10, ZC3H12A, ZFP36, ZNF217, ZNFX1,* | | 683 |  |
| Total | |  | 672^b^ |  |

^a^ Genes were selected based on published literature (PMID: 37433793);

^b^ 11 genes in X chromosome had been removed

| **Supplementary Table 3.** Associations of the first 10 principal components and OS of NSCLC in the PLCO trial | | | | |
| --- | --- | --- | --- | --- |
| **PC*** | **Parameter Estimate** | **Standard Error** | **Chi-Square** | ***P*** |
| **PC1** | **4.821** | **1.353** | **12.697** | **<0.001** |
| **PC2** | **-0.681** | **1.228** | **0.308** | **0.579** |
| **PC3** | **-3.054** | **0.949** | **10.351** | **0.001** |
| **PC4** | **-2.837** | **1.246** | **5.184** | **0.023** |
| PC5 | -0.910 | 1.232 | 0.546 | 0.460 |
| PC6 | 1.355 | 1.252 | 1.172 | 0.279 |
| PC7 | -0.236 | 1.218 | 0.038 | 0.846 |
| PC8 | -1.684 | 1.322 | 1.622 | 0.203 |
| PC9 | -1.886 | 1.267 | 2.216 | 0.137 |
| PC10 | 0.347 | 1.240 | 0.078 | 0.180 |
| * The first four PC were used for adjustment for population stratification in the multivariate analysis.  Abbreviations: OS, overall survival; NSCLC, non-small cell lung cancer; PLCO, the Prostate, Lung, Colorectal and Ovarian Cancer Screening Trial; PC, principal component. | | | | |

**Supplementary Table 4.** Function prediction for the 20 Validated SNPs

| **SNP** | **Gene** | **Chr** | **Genotyped** | **RegDB ^a^** | **Haploreg v4.2 ^b^** | | | | | |
| --- | --- | --- | --- | --- | --- | --- | --- | --- | --- | --- |
|  |  |  |  |  | **Promoter histone marks** | **Enhancer histone marks** | **DNAse** | **Motifs changed** | **Selected eQTL hits** | **dbSNP func annot** |
| **rs10493829** | ***LRRC8C*** | **1** | **Yes** | **6** | -- | -- | -- | **9 altered motifs** | **9 hits** | **intronic** |
| rs11119975 | *ATF3* | 1 | No | 5 | -- | -- | -- | -- | 3 hits | intronic |
| rs13004065 | *SP110* | 2 | No | 4 | BLD, BRN, GI | 12 tissues | -- | Irf | 2 hits | intronic |
| rs4542839 | *SP110* | 2 | No | 7 | -- | -- | -- | -- | 1 hit | intronic |
| rs1522171 | *FOXP1* | 3 | No | 6 | -- | GI, LIV | -- | Sox | -- | intronic |
| rs9819895 | *FOXP1* | 3 | No | 5 | -- | IPSC, BLD | GI | Ascl2 | -- | intronic |
| rs2568847 | *FOXP1* ^c^ | 3 | No | 7 | -- | BLD, BRN | BLD | -- | -- | intronic |
| rs12487457 | *FOXP1* | 3 | No | 5 | -- | BLD | -- | 4 altered motifs | -- | intronic |
| rs2237710 | *MET* | 7 | Yes | 5 | -- | SKIN, MUS | -- | 4 altered motifs | 1 hit | intronic |
| rs7867814 | *PLIN2* ^d^ | 9 | No | 4 | -- | 6 tissues | -- | 4 altered motifs | 3 hits | intronic |
| **rs2239193** | ***OAS2*** | **12** | **Yes** | **3a** | **--** | **BLD** | **BLD, BLD** | -- | 2 hits | intronic |
| rs9559788 | *COL4A2* | 13 | No | 5 | 4 tissues | 14 tissues | 8 tissues | -- | -- | intronic |
| rs7328731 | *COL4A2* | 13 | Yes | 5 | -- | 12 tissues | MUS | 4 altered motifs | 2 hits | intronic |
| rs2281973 | *COL4A2* | 13 | Yes | 5 | -- | 8 tissues | -- | Foxl1, Foxq1, ZEB1 | -- | intronic |
| rs2004853 | *COL4A2* | 13 | No | 5 | -- | 4 tissues | ESDR | HMG-IY | -- | intronic |
| rs12432304 | *IRF9* | 14 | No | 5 | -- | BLD | -- | BCL, Irf, NRSF | 6 hits | -- |
| rs17098749 | *PRKCH* | 14 | No | 5 | FAT, GI | 20 tissues | 17 tissues | Ets, NERF1a, p53 | -- | intronic |
| rs10519070 | *RORA* | 15 | Yes | 5 | -- | -- | BLD, BRN | Pou2f2 | -- | intronic |
| rs922782 | *RORA* ^c^ | 15 | Yes | 5 | -- | 4 tissues | -- | Ets, Foxm1, Pou5f1 | -- | intronic |
| **rs3136651** | ***CCL25*** | **19** | **No** | **4** | **GI, THYM, LIV** | **5 tissues** | **GI, GI** | **Ets** | **--** | **intronic** |

Abbreviations: SNP, single nucleotide polymorphism; NSCLC, non-small cell lung cancer; Chr, chromosome; dbSNP func annot, dbSNP function annotation

^a^RegulomeDB: http://regulomedb.org/

^b^ Haploreg: https://pubs.broadinstitute.org/mammals/haploreg/haploreg.php

^c^ *FOXP1* rs2568847 and *RORA* rs922782 were reported in a previous publication (PMID: 35693292).

^d^ *PLIN2* rs7867814 was reported in a previous publication (PMID: 32072637).

| **Supplementary Table 5.** Stratified analysis for associations between unfavorable genotypes and NSCLC survival in the PLCO trial | | | | | | | | | |
| --- | --- | --- | --- | --- | --- | --- | --- | --- | --- |
| **Characteristics** | **0-1 unfavorable genotype ^a^** | **2-3 unfavorable genotype ^a^** | **Multivariate Analysis ^b^ for OS** | | | | **Multivariate Analysis ^b^ for DSS** | | |
|  | **Frequency** | **Frequency** | **HR (95% CI)** | ***P*** | ***P* _inter_ ^c^** | **HR (95% CI)** | | ***P*** | ***P* _inter_ ^c^** |
| Age (years) |  |  |  |  | 0.914 |  | |  | 0.830 |
| ≤ 71 | 439 | 187 | 1.48 (1.18-1.85) | 0.0006 |  | 1.54 (1.22-1.95) | | 0.0003 |  |
| > 71 | 377 | 156 | 1.31 (1.05-1.63) | 0.019 |  | 1.31 (1.04-1.66) | | 0.023 |  |
| Sex |  |  |  |  | 0.0004 |  | |  | 0.0005 |
| Male | 490 | 195 | 1.58 (1.30-1.93) | <0.0001 |  | 1.62 (1.31-2.01) | | <0.0001 |  |
| Female | 326 | 148 | 0.99 (0.76-1.29) | 0.943 |  | 1.04 (0.79-1.35) | | 0.799 |  |
| Smoking status |  |  |  |  | 0.578 |  | |  | 0.737 |
| Never | 74 | 38 | 1.53 (0.86-2.73) | 0.147 |  | 1.61 (0.90-2.87) | | 0.109 |  |
| Current | 284 | 126 | 1.26 (1.97-1.64) | 0.083 |  | 1.30 (0.99-1.72) | | 0.061 |  |
| Former | 458 | 179 | 1.40 (1.14-1.73) | 0.002 |  | 1.44 (1.15-1.79) | | 0.001 |  |
| Histology |  |  |  |  | 0.381 |  | |  | 0.440 |
| Adeno | 395 | 174 | 1.38 (1.10-1.74) | 0.006 |  | 1.42 (1.12-1.80) | | 0.004 |  |
| Squamous | 203 | 76 | 1.19 (0.84-1.69) | 0.326 |  | 1.24 (0.85-1.82) | | 0.271 |  |
| Others | 218 | 93 | 1.30 (0.97-1.73) | 0.077 |  | 1.32 (0.98-1.79) | | 0.071 |  |
| Tumor stage |  |  |  |  | 0.034 |  | |  | 0.153 |
| I-IIIA | 460 | 187 | 1.27 (0.99-1.63) | 0.061 |  | 1.37 (1.04-1.80) | | 0.025 |  |
| IIIB-IV | 356 | 156 | 1.41 (1.15-1.72) | 0.001 |  | 1.44 (1.17-1.77) | | 0.0006 |  |
| Chemotherapy |  |  |  |  | 0.198 |  | |  | 0.094 |
| No | 428 | 202 | 1.41 (1.13-1.77) | 0.003 |  | 1.49 (1.17-1.89) | | 0.001 |  |
| Yes | 388 | 141 | 1.33 (1.06-1.66) | 0.012 |  | 1.35 (1.08-1.70) | | 0.009 |  |
| Radiotherapy |  |  |  |  | 0.782 |  | |  | 0.712 |
| No | 522 | 227 | 1.41 (1.15-1.74) | 0.0009 |  | 1.46 (1.17-1.82) | | 0.0007 |  |
| Yes | 294 | 116 | 1.33 (1.04-1.71) | 0.024 |  | 1.36 (1.05-1.75) | | 0.019 |  |
| Surgery |  |  |  |  | 0.647 |  | |  | 0.430 |
| No | 439 | 186 | 1.27 (1.06-1.53) | 0.011 |  | 1.31 (1.08-1.58) | | 0.006 |  |
| Yes | 377 | 157 | 1.52 (1.14-2.03) | 0.005 |  | 1.60 (1.16-2.21) | | 0.005 |  |
| Abbreviations: OS, overall survival; DSS, disease-specific survival; NSCLC, non-small cell lung cancer; PLCO, the Prostate, Lung, Colorectal and Ovarian Cancer Screening Trial; HR, hazards ratio; CI, confidence interval.  ^a^ 26 with missing data were excluded; Unfavorable genotypes were *LRRC8C* rs10493829 TT, *OAS2* rs2239193 AG+GG, and *CCL25* rs3136651 TT.  ^b^ Adjusted for age, sex, stage, histology, smoking status, chemotherapy, radiotherapy, surgery, PC1, PC2, PC3 and PC4;  ^c^ *P* _inter_: *P* value for interaction analysis between characteristics and unfavorable genotypes. | | | | | | | | | |

**Supplemental Acknowledgements**

We wish to thank all of the investigators and funding agencies that enabled the deposition of data in dbGaP and PLCO that we used in the present study:

The datasets used for the analyses described in thr present study were obtained from dbGaP at http://www.ncbi.nlm.nih.gov/gap through dbGaP accession number phs000336.v1.p1 and phs000093.v2.p2. Principal Investigators: Maria Teresa Landi. Genetic Epidemiology Branch, Division of Cancer Epidemiology and Genetics, National Cancer Institute, National Institutes of Health, Bethesda, MD, USA. Neil E. Caporaso. Genetic Epidemiology Branch, Division of Cancer Epidemiology and Genetics, National Cancer Institute, National Institutes of Health, Bethesda, MD, USA.

Funding support for the GWAS of Lung Cancer and Smoking was provided through the NIH Genes, Environment and Health Initiative [GEI] (Z01 CP 010200). The human subjects participating in the GWAS derive from The Environment and Genetics in Lung Cancer Etiology (EAGLE) case-control study and the Prostate, Lung Colon and Ovary Screening Trial and these studies are supported by intramural resources of the National Cancer Institute. Assistance with phenotype harmonization and genotype cleaning, as well as with general study coordination, was provided by the Gene Environment Association Studies, GENEVA Coordinating Center (U01HG004446). Assistance with data cleaning was provided by the National Center for Biotechnology Information. Funding support for genotyping, which was performed at the Johns Hopkins University Center for Inherited Disease Research, was provided by the NIH GEI (U01HG004438).

PLCO was also supported by the Intramural Research Program of the Division of Cancer Epidemiology and Genetics and by contracts from the Division of Cancer Prevention, National Cancer Institute, NIH, DHHS. The authors thank PLCO screening center investigators and staff, and the staff of Information Management Services Inc. and Westat Inc. Most importantly, we acknowledge trial participants for their contributions that made this study possible.
